# Mendelian randomization analysis of the causal impact of body mass index and waist-hip ratio on rates of hospital admission

**DOI:** 10.1016/j.ehb.2021.101088

**Published:** 2022-01

**Authors:** Audinga-Dea Hazewinkel, Rebecca C. Richmond, Kaitlin H. Wade, Padraig Dixon

**Affiliations:** a Population Health Sciences, Bristol Medical School, University of Bristol, UK; b MRC Integrative Epidemiology Unit, Bristol Medical School, University of Bristol, UK; c Nuffield Department of Primary Care Health Sciences, University of Oxford, UK

**Keywords:** Body mass index, BMI, Waist hip ratio, WHR, Hospital admissions, Mendelian randomization, Instrumental variables, Poisson models

## Abstract

We analyze how measures of adiposity – body mass index (BMI) and waist hip ratio (WHR) – causally influence rates of hospital admission. Conventional analyses of this relationship are susceptible to omitted variable bias from variables that jointly influence both hospital admission and adipose status. We implement a novel quasi-Poisson instrumental variable model in a Mendelian randomization framework, identifying causal effects from random perturbations to germline genetic variation. We estimate the individual and joint effects of BMI, WHR, and WHR adjusted for BMI. We also implement multivariable instrumental variable methods in which the causal effect of one exposure is estimated conditionally on the causal effect of another exposure. Data on 310,471 participants and over 550,000 inpatient admissions in the UK Biobank were used to perform one-sample and two-sample Mendelian randomization analyses. The results supported a causal role of adiposity on hospital admissions, with consistency across all estimates and sensitivity analyses. Point estimates were generally larger than estimates from comparable observational specifications. We observed an attenuation of the BMI effect when adjusting for WHR in the multivariable Mendelian randomization analyses, suggesting that an adverse fat distribution, rather than a higher BMI itself, may drive the relationship between adiposity and risk of hospital admission.

## Highlights

• Adiposity has a causal effect on increased hospital admission risk.
• Effect of body mass index on hospital admission rate is mediated by waist-hip-ratio.
• Effect of adiposity on hospital admissions is driven by detrimental fat distribution.
• First Mendelian randomization study of adiposity on all-cause hospital admissions.
• First Mendelian randomization study with Poisson model for gene-outcome association.

## 1 Introduction

Individuals with higher adiposity, as indexed by measures such as body mass index (BMI) and waist hip ratio (WHR), attend hospital more frequently than others (Buys et al., 2014, Chen et al., 2007, Han et al., 2009, Korda et al., 2015, Migliore et al., 2013, O'Halloran et al., 2020, Reeves et al., 2014). Establishing the causal impact of adiposity on hospital admissions is an important step in understanding the impacts of adverse weight profiles on the health system. This importance stems from a number of considerations.

In the first instance, BMI (a marker of overall body fat) and WHR (a marker of regional adiposity) are associated with increased incidence of various diseases (Corbin et al., 2016, Dale et al., 2017, Dalton et al., 2003, Folsom et al., 2000, Hu et al., 2007, Lyall et al., 2017, Staiano et al., 2012, Timpson et al., 2009) and all-cause and cause-specific mortality (Srikanthan et al., 2009, Staiano et al., 2012, Wade et al., 2018). Moreover, the incidence of adverse adiposity profiles is also increasing across the world. World Health Organization statistics identified 39% of men and 40% of women as overweight (BMI>25 kg/m^2^) and 11% of men and 15% of women as obese (BMI>30 kg/m^2^) worldwide (World Health Organization, 2016). Additionally, a positive association between BMI and healthcare costs has been identified (Cawley, 2015a; Dixon, Davey Smith, and Hollingworth, 2019; Dixon, Hollingworth, Harrison, Davies, and Davey Smith, 2020; Finkelstein, 2011; Kent et al., 2017a; Kent et al., 2017b; Withrow and Alter, 2011). However, observational assessments of the association between adiposity and hospital attendance are challenged by endogeneity attributable to unobserved confounding and reverse causation, precluding accurate causal inference (Auld and Grootendorst, 2011; Cawley, 2015a, Cawley, 2015b; Cawley and Meyerhoefer, 2012).

Bias due to reverse causality occurs when the outcome of interest (e.g., hospital admissions) also affects the exposure (e.g., BMI). For example, obesity-induced disease resulting in hospital admission could lead to weight loss. This could bias the association between adiposity and hospital attendance towards the null, or, in the most extreme case, result in the conclusion that weight loss leads to hospital admission (Flanders and Augestad, 2008). Bias due to confounding occurs when a common cause of the exposure and outcome exists and, if measured or unmeasured, remains unaccounted for in the model. For example, unobserved or inadequate measures of socioeconomic status may confound the association between adiposity and hospital admissions, with the observational estimate overstating the true effect. Asaria et al. examined hospital episode statistics in the UK and found that lower socioeconomic status was associated with greater health-care needs and more frequent hospital admissions (Asaria, Doran, and Cookson, 2016), while previous studies found that lower socioeconomic status resulted in an on average higher BMI and a greater risk of obesity (Adler and Ostrove, 1999, Tyrrell et al., 2016).

In this paper, we introduce the first Mendelian randomization analysis to use hospital admissions as an outcome. We used UK Biobank data from over 300,000 adults aged 39–72, with over 550,000 in-patient hospital admissions in relation to three related exposures: BMI, WHR and WHR adjusted for BMI (WHRadjBMI). Mendelian randomization (MR) is an instrumental variable approach that permits the robust estimation of the causal effect of an exposure or treatment variable (e.g., BMI) on an outcome (e.g., hospital admissions) (Davey Smith and Hemani, 2014; Haycock et al., 2016). The identifying assumption of MR is the quasi-random allocation of genetic variants from parents to their children that occurs at conception. Elements of this variation are known to associate with traits such as BMI and WHR, and may be used as instrumental variables in causal analyses relating the effects of these adiposity-related exposure variables to hospital admission outcomes.

MR reduces or eliminates problems of confounding and other types of bias common to conventional studies of the associations between measures of adiposity and healthcare-related resource use. For example, MR can rule out reverse causation, since germline variants are determined at conception, and, in principle, should not be affected by confounding from unmeasured variables, given the quasi-random allocation of variants from parents to offspring at conception.

We employed three broad classes of estimator to assess these causal relationships. We first implemented a generalized linear model (GLM) version of the familiar two-stage least squares (2SLS) estimator. Our outcome was hospital admission counts for subjects observed for varying lengths of time, reflecting the duration of follow-up available in our outcome data, while accounting for time of recruitment and censoring due to end of follow-up or death. As Poisson regression models are linear on the logarithmic scale, the second stage of the standard 2SLS instrumental variable estimator was replaced by a Poisson regression. These models were just identified, utilizing a genetic risk score (a single summary measure indicating genetic liability to the exposure of interest) as a single instrumental variable.

Valid instrumental variables are associated with the exposure of interest, are conditionally independent of known and unknown omitted variables, and affect the outcome only via their effect on the exposure (the exclusion restriction). The most likely source of violation of these assumptions in MR is the exclusion restriction, and for that reason a variety of estimators were employed to test the robustness of our results to the presence and consequences of any violations of this assumption. The second broad class of estimators therefore involved over-identified models that allow that exclusion restriction to be relaxed for some or all variants, at the cost of other assumptions that we set out below.

Finally, in addition to testing the direct, individual effect of these exposures on admissions, we also implemented multivariable instrumental variable models, in which the causal effect on one exposure is estimated conditional on the causal effect of another exposure. This permits evaluation of whether the causal effect of one exposure on admissions is mediated by another exposure. For example, this approach allows for estimation of the direct effect of BMI on hospital admissions that is not mediated via the indirect effect of BMI on WHR, and (simultaneously) the direct of effect of WHR that is not mediated by the indirect effect of BMI on this outcome.

Our work extends related analyses of the causal effect of BMI and body fat percentage on healthcare costs (Dixon et al., 2020). The Dixon et al. paper established that higher BMI is associated with higher hospital costs, and that this effect did not appear to be mediated by body fat percentage. This new paper aims to make at least four additional contributions. The first is to interrogate a mechanism that may underlie this association. It is not necessarily obvious that the relationship between adiposity and hospital admissions would be the same as the relationship between adiposity and costs, since it is possible that adiposity does not increase rates of admission. The second is to examine the impact of a further adiposity-related exposure (WHR) as a means of testing whether regional as well as overall adiposity may contribute to rates of admission. The third is to apply, for the first time, Mendelian randomization (MR) as a robust method for causal inference to the literature on the association between adiposity and admissions. This literature is important for the development of inputs into quantitative models that evaluate healthcare policies and assess the cost-effectiveness of interventions intended to modulate rates of hospital admission in relation to BMI and other measures of adiposity. Finally, we introduce, for the first time, a quasi-Poisson instrumental variable framework for analyzing a rate outcome in an MR study, which will support efforts to model similar rate outcomes in other contexts.

The rest of the paper is set out as follows. We introduce the data used for our analysis before describing in detail our methods for estimating the direct and indirect effect of these exposures on admissions. We conclude with our interpretation of these results. Our results indicate that the effect of various measures of adiposity on admission may be larger than conventional observational analysis would indicate, and that regional adiposity (as indexed by WHR) may play a particularly important role in influencing the rate of hospital admissions.

## 2 Data

### 2.1 UK Biobank data

The UK Biobank study is a resource of phenotypic, genetic, electronic health record and death registry data, collected from over 500,000 individuals, from 2006 to 2010 (Bycroft et al., 2018, Collins, 2012, Sudlow et al., 2015). Participants were aged 39–72 years at recruitment and were predominantly of White British ethnicity (Sudlow et al., 2015). The UK Biobank received ethical approval from the North West-Haydock Research Ethics Committee (reference 11/NW/0382).

**Fig. 1:**
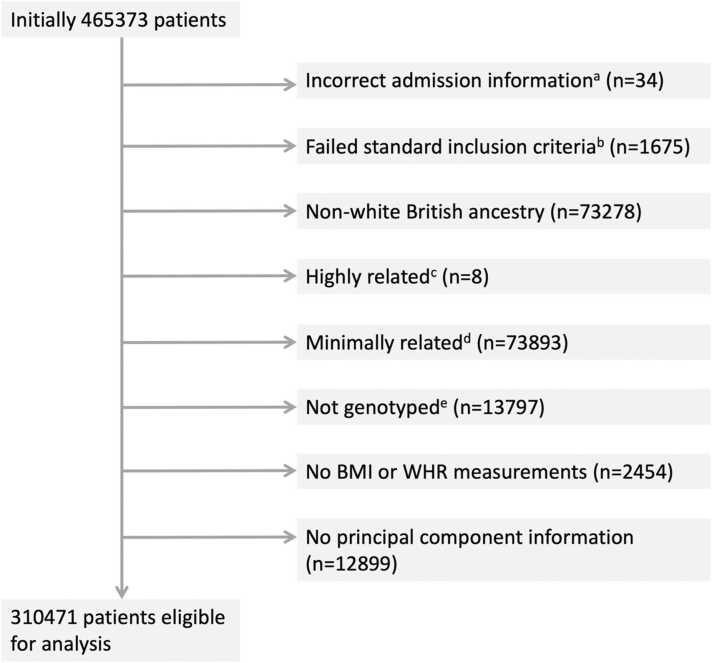
Participant inclusion diagram. Notes: Eight exclusion categories are shown with the corresponding numbers of participants per category. On removal of duplicates a total of 154902 unique participants were considered ineligible for analysis. a) admissions prior to study start date, post death/censoring date or registered death prior to study start; b) individuals that have a mismatch between genetically inferred and reported gender, individuals with sex chromosome types putatively different from XX or XY and individuals that are outliers in heterozygosity and missing rate; c) individuals related to more than 200 other participants; d) on exclusion a maximal set of unrelated individuals is retained; e) not genotyped for the exposures of interest (BMI, WHR, WHRadjBMI).

For 465,373 participants, information on hospital inpatient admissions was available through linked Hospital Episode Statistics (HES) data. From this set, 310,471 participants were considered eligible for analysis (Fig. 1). Participants were removed when admission information was incorrect, when the genetic data did not meet the standard of a documented in-house quality control procedure (Mitchell, 2019), and when BMI and/or WHR measurements were absent. All analyses (both observational and instrumental variable models) were restricted to individuals of White British ancestry to avoid confounding by differential ancestry under the second instrumental variable assumption. Participants matching one or more of the exclusion criteria were removed (N = 154,902). An overview of all relevant exclusion criteria is given in the participant exclusion diagram in Fig. 1, with Table S1 providing a cross-table overview of participant overlap for any two exclusion criteria.

### 2.2 Adiposity measures

Measures on weight, bio-impedance, height, waist circumference and hip circumference were collected at the baseline UK Biobank recruitment assessment. Weight and bio-impedance were measured using the Tanita BC-418MA body composition analyzer. Standing height was measured using a Seca 202 height measure. BMI was calculated as weight divided by height squared (kg/m^2^), and using electrical impedance. When the first measure was unavailable, values were supplemented with the latter. WHR was calculated by dividing waist circumference by hip circumference, measured with a Wessex non-stretchable spurring tape measure (UK Biobank, 2011). BMI and WHR values reflect baseline measures of adiposity when individuals were recruited into UK Biobank, which occurred on a rolling basis from 2006 to 2010. They do not reflect intertemporal variation in adiposity. Our causal Mendelian randomization estimates reflect the cumulative and long-term impact of quasi-random allocation to genetic liability to higher or lower adiposity over the life course.

### 2.3 Hospital admission counts

For 465,373 participants, information on hospital inpatient admissions ("admitted patient care" or APC episodes) was available through linked Hospital Episode Statistics (HES) data. Inpatient admissions involve a patient occupying a hospital bed for some period, but do not necessarily imply an overnight stay. These data do not include emergency admissions or outpatient care episodes. Our data include all inpatient care that is publicly funded in both NHS and private hospitals, but not privately-funded care in private hospitals. For a given hospital admission, a patient may have multiple “episodes” of care. Admissions were therefore defined when an individual had episodes starting on separate dates, excluding incomplete episodes, episodes with inconsistent or overlapping start and end dates, and accounting for patient transfers. Code to define the admissions variable is available from https://github.com/pdixon-econ/admissions-biobank.

### 2.4 Genetic variants

Estimates for 77 genetic variants associated with BMI at a genome-wide significance level (p < 5 × 10^-8^) in the largest genome-wide association study meta-analysis of a combined number of up to 322,154 individuals of European descent (not including UK Biobank) were obtained from the Genetic Investigation of Anthropometric Traits (GIANT) consortium (Locke et al., 2015). Individual-level genetic data of sufficient quality (Mitchell, 2019) was available from UK Biobank for 76 of these 77 SNPs. Genetic variants associated with WHR and WHRadjBMI were also obtained from the GIANT consortium, with 39 and 48 SNPs, respectively, identified in relation to WHR at p < 5 × 10^-8^, in a meta-analysis of up to 210,088 individuals (Shungin et al., 2015).

## 3 Methods

### 3.1 Genetic variants as instrumental variables

Many introductions to MR are available both in general (Davey Smith and Ebrahim, 2003; Haycock et al., 2016; Pingault et al., 2018) and in relation to health economic outcomes (Dixon et al., 2019; Dixon, Davey Smith, von Hinke, Davies, and Hollingworth, 2016; Harrison et al., 2020; von Hinke Kessler Scholder et al., 2011; Von Hinke et al., 2016). Here we briefly review the instrumental variable assumptions in the context of MR.

Certain parts of the genome are subject to variation between individuals in a population. At each of point variation, offspring inherit an allele – the specific form of genetic variation – from each of their parents according to Mendel’s first and second laws of inheritance. Mendel’s first law describes random segregation of alleles from parents to offspring. Mendel’s second law describes the independent assortment for different traits of these alleles. Together, the two laws imply that offspring have an equal chance of inheriting an allele from either parent, and that these alleles are inherited independently from one another. The allocation of these variants is therefore random, conditional on parental genomes.

It is this form of conditionally random allocation and its use as an identification mechanism in instrumental variable analysis that is known as Mendelian randomization (Davey Smith and Ebrahim, 2003). MR may therefore be interpreted as a type of natural experiment, in which individuals or groups of individuals are allocated to groups indicating higher or lower genetic liability to (for example) higher BMI or WHR. Mendelian randomization meets the definition of natural experiments used in economics, such as in Di Nardo (DiNardo, 2008), in the sense that they refer to serendipitous random assignment of individuals to a treatment or a control group, and outcomes may be analyzed in relation to this assignment. Under the assumptions of instrumental variable analysis, the quasi-random allocation to genetic variation indicating higher or lower levels of adiposity can be used to make causal claims about the effect of these types of exposures on hospital admissions.

We study single nucleotide polymorphisms (SNPs), which are one form of genetic variation (amongst others) that are subject to inheritance under Mendel’s first and second laws. A SNP refers to single change in one of the nucleotides that make up the code of the genome. Nucleotides in DNA are in turn made up in part of the nucleobases (adenine (A), cytosine (C), guanine (G) or thymine (T) which comprise this code. A SNP will therefore involve a substitution of one of these “letters” in the genetic code for another. The possible versions of the SNP at a specific point are the alleles for that location in the genome. Some SNPs are associated with the expression of particular traits or phenotypes, including several adiposity-related phenotypes such as BMI and WHR.

The association of SNPs with phenotypes, together with their conditionally random allocation from parents to offspring, indicate the potential for their analysis as instrumental variables. Humans are diploid, meaning that they have two copies of each chromosome. We may therefore treat SNPs as count variables – humans may have a SNP on both chromosomes (n = 2), only on one (n = 1) or on neither chromosome (n = 0).

Valid instrument variables are associated with the exposure of interest, are independent of all confounding omitted variables (whether measured or unmeasured) and affect the outcome only via the exposure of interest. We briefly unpack these requirements in relation to MR.

Relevance – the requirement that instruments are not independent of exposures – can be determined from genome wide association studies (GWASs), which trawl the genome for signals of association between a predefined exposure and regions of potential genetic variation (McCarthy et al., 2008). Replicated evidence of association from large, well-powered GWASs are the most robust means of establishing the relevance criterion for specific SNPs. In this study, we only use SNPs that meet the relevance requirement for BMI and waist hip ratio.

The second requirement, that of independence from confounding omitted variables, is sometimes interpreted as the requirement that the instrument be “as good as randomly assigned” (Angrist and Pischke, 2009). As genetic variation is determined at conception, it necessarily occurs before many later life circumstances and events such as socioeconomic status, education level, and the local environment. This ensures independence from most potential confounding variables.

Nevertheless, there are a few different means by which this assumption might be violated. Events that are connected with the time of conception, such as year of birth and sex, may confound this association. This requirement will also be violated if there are differences in subgroups defined by allele frequencies that also differ in disease or trait susceptibility. For example, variants in the FTO gene (the first gene to be associated with obesity) confer an increased risk of obesity, but the prevalence of these variants differs significantly by ancestry (Loos and Yeo, 2014). Environments are not necessarily the same between groups of different ancestry. Allele subgroups may also become correlated with the environment for other reasons. For instance, assortative mating describes the mating of genetically similar individuals. Over time, this will tend to lead to a non-random clustering of alleles, potentially violating this assumption.

The third requirement for instrumental variable analysis, the exclusion restriction, may be violated through two principal mechanisms in MR analysis. The first is via so-called linkage disequilibrium, which refers to the fact that SNPs in close physical proximity tend to be inherited together. Use of one of these SNPs may therefore also reflect the effect of other SNPs not intended to be included in the analysis. For example, it may be the case that an adiposity-related SNP is in linkage disequlibrium with a SNP associated with depression. If this linkage disequilibrium is not accounted for, analysis of the adiposity-related SNP will unintentionally also include any impact of the depression-related SNP.

The second and more challenging potential violation of this assumption is via pleiotropy (Hemani et al., 2018a). Pleiotropy is the effect of a genetic variant on more than one phenotype. The exclusion restriction will be violated if a SNP associated with (for example) BMI also affects the outcome through a BMI-independent channel. For example, if a SNP influences both BMI and depression, and the impact of depression is not related to BMI, then analysis of the impact of BMI on the outcome will be confounded by this separate channel of influence of depression on the outcome. This is known as horizontal pleiotropy (Davey Smith and Hemani, 2014). The exclusion restriction will not be violated if the other phenotype does not affect the outcome, or if the other phenotype is an intermediate between the exposure of interest and the outcome (the latter is known as vertical pleiotropy).

### 3.2 Poisson modeling of admissions data in conventional and instrumental variable models

This section describes our approach to modeling the association between hospital admissions and the three measures of adiposity. We employed both conventional observational models and instrumental variable models. Both types of model implemented versions of Poisson regression.

#### 3.2.1 Poisson modeling of admissions data

A Poisson regression model applies a generalized linear model with a logarithmic link function under the assumption that the response variable is Poisson distributed and that the logarithm of the expected value *μ* can be expressed in a linear combination of *k* parameters (Eq. 1).

(1) $$\log\mu =\beta_{1}*x_{1}+\beta_{2}*x_{2}+\ldots +\beta_{k}*x_{k}$$

UK Biobank participants were recruited between 2006 and 2010 and, for each participant, hospital admissions were counted from recruitment to study censoring, with the latter given by either death or 31 March 2015, the date at which the linked Hospital Episode Statistic data were censored for this analysis. We ignored emigration, which is estimated to occur at low rates (0.3%) in this cohort (Fry et al., 2017).

To correct for the varying times on study, the logarithm of observed person-years *t* is added to the linear equation as an offset (Eq. 2).

(2) $$\log\mu =\log t+\beta_{1}*x_{1}+\beta_{2}*x_{2}+\ldots +\beta_{k}*x_{k}$$

The modeled outcome can now be interpreted as a rate rather than a count, as becomes apparent when restructuring Eq. (2) to Eq. (3).

(3) $$\log\frac{\mu }{t}=\beta_{1}*x_{1}+\beta_{2}*x_{2}+\ldots +\beta_{k}*x_{k}$$

Let *x*_*1*_ and β_1_ represent the exposure BMI and the corresponding parameter estimated from the Poisson regression. Then with *t* in years and BMI in kg/m^2^, the exponent of the coefficient exp(β_1_) is the factor by which the mean value of the outcome is multiplied for a 1 kg/m^2^ increase in BMI. With the remaining variables are held constant, the yearly hospital admission rate increases with a factor *exp (β*_*1*_*)*^*n*^ for every *n* unit increase in BMI. Values of the coefficient > 1 indicate an increase in admission rate and values < 1 indicate a decrease.

#### 3.2.2 Multivariable observational analyses

Observational estimates were obtained by regressing each exposure on the outcome in a conventional (i.e. without instrumental variables) Poisson regression, with time in UK Biobank as the offset. Unadjusted estimates were obtained alongside estimates adjusted for age at study entry, sex, and for an additional five categorical variables: alcohol frequency (ranging from never to daily or almost daily), days of exercise per week (ranging from 0 days to 7 days), educational/ professional qualifications, employment (with 57% employed and 35% retired), material deprivation as measured by the Townsend deprivation index (divided into quintiles, with higher values indicating a greater degree of deprivation). To make full use of the available data, we performed multiple imputation, which assumes that missing values are missing at random, conditional on all covariates included in the imputation model. We used a 10-fold imputation approach and pooled the resulting coefficients and standard errors using Rubin’s rules (Rubin, 1996).

#### 3.2.3 Instrumental variable Poisson models

MR methods are traditionally applied to continuous or binary outcome data, whereas count data such as hospital admission data is frequently modeled using Poisson regression. Here, the outcome was given by hospital admission counts for subjects that were observed for varying lengths of time. As the Poisson model is linear on the logarithmic scale, the second stage of the MR regression, which estimates the gene-outcome association, can be replaced by a Poisson regression.

We conducted both one- and two-sample MR analyses. In a one-sample framework, a single sample of individual-level genetic and phenotypic data may be used to obtain estimates of both the gene-exposure association (*β*_*exp*_) and the gene-outcome association (*β*_*out*_). External weightings from the GIANT consortium were used for the gene-exposure associations.

The ratio of the coefficients (the Wald ratio) gives the causal IV estimate: $\beta_{\mathrm{IV}}=\frac{\beta_{\mathrm{out}}}{\beta_{e\mathrm{xp}}}$. When a single instrument is used in a linear model, the Wald ratio is identical to the two-stage least squares (2SLS) estimate. The first stage constitutes a linear regression of the exposure on the instrument (gene), while the second stage regresses the outcome on the predicted values obtained from the first regression. When the linear regression in the second stage is replaced by some non-linear regression, this method of estimation is referred to as a two-stage predictor substitution (2SPS). When the second stage regression has an identity link, as is the case for linear regression, or a collapsible link, like the Poisson log link, 2SPS is equivalent to the Wald ratio approach (Palmer et al., 2011). Here, we use the Wald ratio approach to obtain the causal IV estimate, using a linear regression to obtain *β*_*exp*_ and a Poisson regression to obtain *β*_*out*_.

Note that the numerator and the denominator in the Wald ratio need not come from the same sample (Angrist and Krueger, 1992). In a two-sample MR framework, the exposure and outcome coefficients may be obtained from separate, independent samples from similar populations (Davey Smith and Hemani, 2014; Haycock et al., 2016). This approach may offer better efficiency than a one-sample approach if larger sample sizes are available when using data from more than one sample. A further important advantage is that the two-sample approach in particular facilitates methods for testing the sensitivity of results to possible violations of the exclusion restriction, as we discuss below.

We employed one- and two-sample MR models. For both the one- and two-sample analyses we implemented a model where the second stage linear regression of the outcome, *Y*_*adm*_ , on the respective SNPs is replaced by a Poisson regression with the person years on study, *t,* as offset. Let β_exp_ be the exposure coefficient obtained from a linear regression of the exposure, *Y*_*exp*_ , on the genetic instrument or risk score, *G* (Eq. 4)*,* and β_out_ the outcome coefficient on the logarithmic scale obtained from a Poisson regression of the outcome, *Y*_*out*_ , on the genetic instrument, *G* (Eq. 5)*.*

(4) $$Y_{\exp}\sim \beta_{\exp}*G$$

(5) $$\log\left({E[Y}_{\mathrm{out}}]\right)\sim \log\left(t\right)+\beta_{\mathrm{out}}*G$$

As the Poisson model is linear on the log scale, the two coefficients are compatible and a valid ratio can be obtained, with the final IV estimate, β_IV_ , of the rate coefficient given by the exponent of this ratio (Eq. 6).

(6) $$\beta_{\mathrm{IV}}=\exp(\frac{\beta_{\mathrm{out}}}{\beta_{\exp}})$$

The coefficient $\beta_{\mathrm{IV}}$ is the factor by which the yearly hospital admission rate increases for each unit of exposure, again with values > 1 indicating an increase in admission rate and values < 1 indicating a decrease.

The Poisson model assumes an outcome distribution such that the outcome mean is equal to the outcome variance. As the hospital admission count variance (55.3) was greater than the mean hospital admission count (1.89), we used a quasi-Poisson model instead (Ver Hoef and Boveng, 2007). Standard errors for the causal IV estimates were estimated using Taylor series expansions (Thomas, Lawlor, and Thompson, 2007).

#### 3.2.4 One-sample just-identified Mendelian randomization

Weighted genetic risk scores (GRS)s were constructed for BMI (76 SNPs) (Locke et al., 2015), WHR (39 SNPs) (Shungin et al., 2015) and WHRadjBMI (48 SNPs) (Shungin et al., 2015). To ensure a meaningful interpretation of the score, the exposure-increasing allele for each genetic variant was chosen as the effect allele (Wade et al., 2018). For every study participant, the dosage for each relevant genetic variant was extracted from the UK Biobank genetic data and weighted with the effect size reported by the GIANT consortium. Following this, the weighted dosages were summed and divided by the sum of all effect sizes, giving, for each exposure, a single GRS representing an estimate of the average number of exposure-increasing alleles. Estimates were adjusted for age, sex and the first 40 genetic principal components (PCAs) in order to comply with the second instrumental variable assumption of conditional independence (“as good as randomly assigned”). To estimate the effect of WHRadjBMI on hospital admissions, we used the residuals of a regression of WHR on BMI as an exposure, which gave an estimate for the predictive performance of the WHR component that cannot be linearly predicted by BMI. Additionally, we investigated the effect of the exposures BMI, WHR and WHRadjBMI separately in male and female study participants.

We also considered the effects of BMI and WHR when estimated in a joint model, using a multivariable MR approach. Multivariable MR aims to estimate the causal effect of multiple exposures simultaneously. In contrast to univariable MR, which estimates the total effect of an exposure on the outcome, Multivariable MR estimates the direct effect of each exposure, conditioning on the causal effects of the SNPs on the other exposure (Sanderson, Davey Smith, Windmeijer, and Bowden, 2019). We regressed BMI and WHR separately on the full combined set of SNPs and regressed the fitted values of both on hospital admission count in a quasi-Poisson regression with time as the offset. Standard errors were obtained through a 10,000-fold full-sample bootstrap (Efron and Tibshirani, 1993).

For BMI, estimates of hospital admission rates per year were obtained per BMI unit (1 kg/m^2^) and per BMI standard deviation (SD). For WHR, WHRadjBMI, and the WHR residuals, estimates were obtained per 0.10 WHR unit and per WHR SD. The relevant SDs were calculated directly from the UK Biobank data, yielding SDs of 4·74 kg/m^2^ and 0.09 for BMI and WHR, respectively.

#### 3.2.5 Two-sample over-identified summary Mendelian randomization

We employed a variety of over-identified methods to assess the causal relationship between adiposity and hospital admissions in two-sample MR analyses. Four two-sample MR approaches were used to investigate the effect of BMI, WHR and WHRadjBMI on yearly hospital admission rate: (1) the random effects exact weights inverse-variance weighted (IVW) estimator (Bowden et al., 2019); (2) the random effects Mendelian randomization Egger (MR-Egger) estimator (Bowden, Davey Smith, and Burgess, 2015); (3) the penalized median estimator (Bowden, Davey Smith, Haycock, and Burgess, 2016); (4) the weighted mode estimator (Hemani et al., 2018a).

All these approaches make distinct assumptions about if and how the exclusion restriction might be violated. Precise technical details are available in the respective references. Here, we provide an overview of these details and some intuition for their implementation.

A useful starting point is to approach this type of instrumental variable analysis from the perspective of meta-analysis (Bowden and Holmes, 2019). If each SNP is treated as the outcome of a natural experiment occurring at conception, then an overall effect estimate across many SNPs may be obtained by performing meta-analysis. For example, for the random effects models, Wald ratios are estimated for each SNP separately and combined using a random effects meta-analysis approach. The traditional IVW estimator uses weights derived from the inverse variance of the SNP-outcome coefficient. Let *β*_*j*_ be the Wald ratio estimate for some SNP, *j*, given by the ratio of the SNP-outcome association over the SNP-exposure association, estimated by:

(7) $${\hat{\beta }}_{j}\text{=}\frac{{\hat{\beta }}_{Y_{j}}}{{\hat{\beta }}_{X_{j}}}.$$

Then, the IVW estimate is given by

(8) $${\hat{\beta }}_{\mathrm{IVW}}=\frac{\sum_{j}{\hat{\beta }}_{X_{j}}^{2}\sigma_{Y_{j}}^{-2}{\hat{\beta }}_{j}}{\sum_{j}{\hat{\beta }}_{X_{j}}^{2}\sigma_{Y_{j}}^{-2}},$$

with $\sigma_{Y_{j}}^{2}$ the variance of the SNP-outcome association. The exact weights IVW estimator derives weights in a slightly different way, using a limited information maximum-likelihood (LIML) approach in which the weight term is allowed to be a function of the causal-effect parameter. This approach therefore also ensures the estimator is naturally robust against regression-dilution bias (Bowden et al., 2019).

This approach assumes either that there is no horizontal pleiotropy in violation of the exclusion restriction, or that any horizontal pleiotropy balances out such that the effect point estimates are not biased. Violations of the exclusion restriction induced by horizontal pleiotropy may be apparent if the effect of a SNP or set of SNPs is large relative to the mean effect of all SNPs.

We assessed heterogeneity of this type in two related ways. The first was via Cochran’s Q statistic, the two-sample analogue of the Sargan test for overidentification. Heterogeneity can be assessed by comparing the Q statistic (Eq. 9) to the critical values of a chi-squared distribution.

(9) $$Q=\sum_{j=1}^{J}\frac{1}{\sigma_{Y_{j}}^{2}}{\left({\hat{\beta }}_{j}-{\hat{\beta }}_{\mathrm{IVW}}\right)}^{2}$$

This assumes up to *J* SNPs; ${\hat{\beta }}_{j}$ and ${\hat{\beta }}_{\mathrm{IVW}}$ measure the effect estimate for SNP *j* and the overall inverse variance (IVW) weighted effect over all *J* SNPs, respectively. The variance of the SNP-outcome association is given by $\sigma_{Y_{j}}^{2}$.

The second, related means of assessing heterogeneity was based on Rucker’s Q’ statistic (Bowden et al., 2018a), which should be considered in context of the MR-Egger approach. The random effects IVW model constrains the overall IVW-regression line to pass through the origin; if this were not the case, then some or all variants would be violating the exclusion restriction, since the causal effect (absent violations of the exclusion restriction) is determined only by the Wald ratio. The random effects MR-Egger model does not constrain the intercept to zero. The SNP-outcome coefficients are regressed on the SNP-exposure coefficients:

$${\hat{\beta }}_{Y_{j}}\text{=}\beta_{0E}{{+\beta }_{E}\hat{\beta }}_{X_{j}},$$

with all ${\hat{\beta }}_{X_{j}}$ associations oriented to be positive (i.e., the reference allele is chosen such that the resulting association is positive). The regression is weighted with the inverse variances of the SNP-outcome associations, $\sigma_{Y_{j}}^{-2}$. The MR-Egger regression intercept, $\beta_{0E}$, can be interpreted as the average pleiotropic effect of all variants. Verifying whether the observed intercept is statistically different from zero serves as a test for horizontal pleiotropy (Bowden et al., 2015).

In essence, this amounts to testing and then conditioning on any horizontal pleiotropy, at the cost of further assumptions concerning the association between instrument strength and the direct pleiotropic effect of variants – on this assumption, see further detail in (Bowden et al., 2015; Burgess and Thompson, 2017). Rucker’s Q’ statistic may be calculated by estimating the MR-Egger model, adjusting for any mean pleiotropic effect, and then testing if any residual heterogeneity is present.

The third estimator is the median estimator, which is given by the median ratio estimate of ordered Wald ratios. The intuition for this estimator is that a consistent estimate will be obtained from the median estimate if at least 50% of the SNPs are valid; invalid estimates will contribute no weight to the overall estimate, provided that at least this proportion of SNPs are valid. The penalized weighted median estimator will be consistent when at least 50% of the weights come from valid instruments. We report results from the penalized weighted median estimator, in which Cochran’s Q statistic is used to quantify heterogeneity and more heterogeneous, outlying variants are down-weighted (Bowden et al., 2016).

The mode estimate is given by the mode of the Wald ratio estimates and will consistently estimate the causal effect even if more than half of the SNPs are invalid, provided the largest homogeneous cluster of SNPs is valid. Both the weighted median and mode estimators use weights derived from the inverse variance of the ratios of the gene-outcome and gene-exposure association estimates and are, under different assumptions, robust to outliers and invalid instruments (Hemani et al., 2018a).

Gene-exposure association coefficients for the two-sample MR analyses were obtained from the GIANT consortium (Locke et al., 2015, Shungin et al., 2015) and gene-outcome association coefficients from the UK Biobank data (Bycroft et al., 2018, Collins, 2012, Sudlow et al., 2015).

Two-sample MR estimates were obtained for BMI, WHR and WHRadjBMI individually and for BMI and WHR jointly in a multivariable two-sample MR IVW analysis (Sanderson et al., 2019). For BMI, estimates of hospital admission rates per year were obtained per BMI unit (1 kg/m^2^) and SD. For WHR and WHRadjBMI estimates were obtained per 0.10 WHR unit and SD. SDs were calculated by taking the median SD across all studies used to obtain the summary measures, giving SDs of 4·60 kg/m^2^ and 0.07 for BMI and WHR, respectively. Plots of the two Q statistics (Cochran’s Q and Rucker’s Q’) were calculated for each SNP in a leave-one-out analysis and were used for the visual identification of outliers, which were removed in a sensitivity analysis with the purpose of verifying estimator consistency.

A threshold of R^2^ < 0·001 to account for linkage disequilibrium (LD) was employed for the two-sample MR analyses. Post LD-correction, 64, 34 and 45 SNPs were retained for BMI, WHR and WHRadjBMI, respectively. For the multivariable two-sample MR analysis, 70 SNPS were retained after a joint LD adjustment for BMI and WHR.

All analyses were performed in R version 3.6.1 (R Development Core Team, 2014). R packages *TwoSample MR* (Hemani et al., 2018b), *Radial MR* (J. Bowden et al., 2018b), and *MVMR* (Sanderson et al., 2019) were used for the two-sample summary Mendelian randomization analyses, and *Amelia* (Honaker, 2019) was used for the multiple imputation performed in the conventional multivariable analyses. An *R* code appendix documenting all analytical steps taken is provided in the supplementary materials.

## 4 Results

This section sets out the results of the analyses described above. We begin with a summary of descriptive statistics and any missingness in these variables. We then introduce results from our observational analysis, which serve as a useful benchmark against which to judge the MR instrumental variable models. We then present the results from the one-sample MR using the GRS instruments, before considering the over-identified two-sample models that test potential violations of the exclusion restriction. We obtained effect estimates per BMI and WHR unit and per SD. To facilitate comparisons, we only report estimates per SD in the main text and provide per unit estimates in the supplementary material.

### 4.1 Descriptive statistics

The 310,471 participants included in the analysis sample had an average age of 57·40 years (SD: 7·99), with a BMI of 27.38 kg/m^2^ (SD: 4·74) and WHR of 0.87 (SD: 0.09). Of these participants, 53·66% were female. Average follow-up time was 6·05 years (SD: 0.91), with 2.0% of patients dying before the end of study. BMI, WHR, sex and age distributions were comparable across the UK Biobank and the GIANT consortium populations (Bycroft et al., 2018, Locke et al., 2015, Shungin et al., 2015). Demographics for the UK Biobank participants are given in Table 1.

**Table 1:** Patient demographics at baseline for N = 310,471 patients. For continuous variables mean and standard deviation are given, for categorical variables counts and percentages per category.

**Characteristic**	**N / mean (SD)**	**%**	**Characteristic**	**N**	**%**
Age at entry	57.402 (7.988)	100	Qualifications		
Missing	0	0	A levels/AS levels or equivalent	35,268	11.4
Sex			College or University degree	96,670	31.1
Female	166,610	53.7	CSEs or equivalent	17,714	5.70
Male	143,861	46.3	NVQ or HND or HNC or equivalent	20,418	6.60
Missing	0	0	O levels/GCSEs or equivalent	69,815	22.5
BMI	27.385 (4.743)	100	Other professional qualifications (e.g., nursing, teaching)	15,821	5.10
Missing	0	0	Missing	54,765	17.6
WHR	0.872 (0.09)	100	Employment		
Missing	0	0	Doing unpaid or voluntary work	1298	0.40
Alcohol frequency			Full or part-time student	544	0.20
Daily or almost daily	67,391	21.7	In paid employment or self- employed	175,679	56.6
Never	19,900	6.4	Looking after home and/or family	8042	2.60
Once or twice a week	81,161	26.1	Retired	109,314	35.2
One to three times a month	34,254	11.0	Unable to work because of sickness or disability	9043	2.90
Special occasions only	32,382	10.4	Unemployed	4212	1.40
Three or four times a week	75,173	24.2	Missing	2339	0.80
Missing	210	0.10	Townsend deprivation		
Days exercise			1st Quint. (−6.258, −4.014]	62,055	20.0
0 days	110,190	35.5	2nd Quint. (−4.014, −2.945]	62,002	20.0
1 day	42,722	13.8	3rd Quint. (−2.045, −1.683]	62,028	20.0
2 days	47,400	15.3	4th Quint. (−1.683, 0.709]	62,030	20.0
3 days	40,976	13.2	5th Quint. (0.709, 10.588]	62,016	20.0
4 days	19,061	6.10	Missing	341	0.10
5 days	19,800	6.40			
6 days	5758	1.90			
7 days	10,315	3.30			
Missing	14,249	4.60			

BMI = body mass index, SD = standard deviation, WHR = waist hip ratio.

**Fig. 2:**
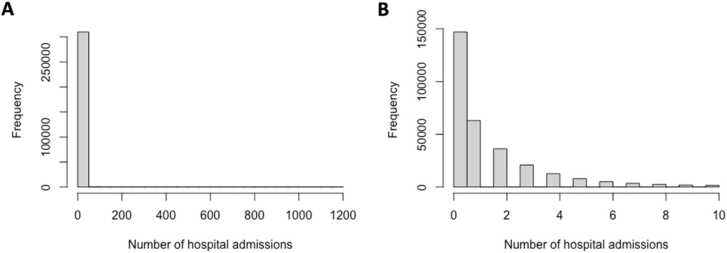
Number of hospital admissions per patient. A) All patients (*N* = 310,471); B) Patients with 10 or less hospital admissions (*N* = 302,254; 97%).

A total of 588,147 in-patient hospital admissions were recorded, with 79% of participants admitted twice or less, and 47% without a single admission. Fig. 2 shows a histogram of the distribution of the number of hospital admissions in all patients (A) and in patients with 10 or fewer admissions (B). Table 2 gives an overview of hospital admission counts across BMI categories, WHR quantiles, age quantiles and gender.

**Table 2:** Hospital admission counts for 310,471 patients per WHR quantiles, age quantiles, BMI categories and across gender. Shown are the 1st quantile, median, 3rd quantile, mean and SD, alongside the number of individuals (*N*, %) per category.

		**Hospital admission count**				
	**N (%)**	**1**^**st**^**quantile**	**median**	**3**^**rd**^**quantile**	**mean**	**SD**
WHR						
1st quant [0.45–0.82)	77,564 (25)	0	0	2	1.55	6.45
2nd quant [0.82–0.87)	78,087 (25)	0	1	2	1.75	6.01
3rd quant [0.87–0.92)	77,267 (25)	0	1	2	1.9	6.9
4th quant [0.92–2.13)	77,553 (25)	0	1	3	2.38	9.78
BMI						
<18.5	1532 (0.5)	0	1	2	2.18	10.9
[18.5–20)	5563 (1.8)	0	0	2	1.63	4.6
[20–22.5)	31,446 (10)	0	0	2	1.54	5.79
[22.5–25)	64,586 (21)	0	0	2	1.68	9.71
[25–27.5)	74,899 (24)	0	1	2	1.78	6.53
[27.5–30)	57,841 (19)	0	1	2	1.96	7.01
[30–35)	53,766 (17)	0	1	2	2.16	6.43
> 35	20,838 (6.7)	0	1	3	2.66	8.33
Age						
1st quant [40–51)	77,608 (25)	0	0	1	1.24	7.41
2nd quant [51–59)	77,608 (25)	0	0	2	1.67	8.2
3rd quant [59–64)	77,633 (25)	0	1	2	2.03	6.13
4th quant [64–73)	77,622 (25)	0	1	3	2.64	7.77
Sex						
Female	166,610 (54)	0	1	2	1.84	7.28
Male	143,861 (46)	0	1	2	1.96	7.62

BMI = body mass index, SD = standard deviation, WHR = waist hip ratio' under the table, as done in the remaining tables.

### 4.2 Observational multivariable analyses

Poisson multivariable regression models provided evidence that BMI and WHR were associated with an increase in yearly hospital admission rate (Tables 3 and 4).

**Table 3:** Observational multivariable analyses of the effect of BMI, WHR, and WHR adjusted for BMI on yearly hospital admission rate per year in UK Biobank participants of White British ancestry (*N* = 310,471). Effect estimates are provided per BMI SD (SD_BMI_=4.74) and per WHR SD (SD_WHR_=0.090).

	**Observational (unadjusted)**	**Observational (adjusted**^**a**^**)**
	Rate^b^ 95%CI	Rate^b^ 95%CI
BMI (SD)	1.148 (1.133–1.163)	1.077 (1.065–1.091)
WHR (SD)	1.197 (1.181–1.214)	1.162 (1.144–1.182)
WHRadjBMI (SD)	1.155 (1.137–1.173)	1.141 (1.120–1.163)
WHR~BMI residuals (WHR SD)^c^	1.156 (1.136–1.176)	1.123 (1.102–1.145)

BMI = body mass index, CI = confidence interval, SD = standard deviation, WHR = waist hip ratio, WHRadjBMI = waist hip ratio adjusted for BMI

a) Adjusted for sex (categorical), age at study entry, alcohol frequency (categorical, from on a daily basis to never), employment (categorical), qualifications (categorical), Townsend deprivation score (categorical in quintiles, where 1 is not deprived and 5 is very deprived), and days of exercise per week (categorical, from 1 to 7). The WHRadjBMI observational analyses also include BMI as a predictor.

b) Estimates (with corresponding 95% CIs) represent the fold increase in yearly hospital admission rate per BMI SD (4.74 kg/m^2^) and per WHR SD (0.090). Estimates per BMI and WHR unit are given in Table S2.

**Table 4:** Observational multivariable analysis of the effect of BMI and WHR jointly on yearly hospital admission rate in UK Biobank participants of White British ancestry (*N* = 310471). Rates and 95% confidence intervals (95% CI) are given. Estimates are provided per exposure SD (SD_BMI_=4.74 and SD_WHR_=0.090).

	**Observational (unadjusted)**	**Observational (adjusted**^**a**^**)**
	Rate^b^ 95% CI	Rate^b^ 95% CI
BMI (SD)	1.084 (1.068–1.100)	1.029 (1.015–1.043)
WHR (SD)	1.155 (1.137–1.173)	1.141 (1.120–1.163)

BMI = body mass index, CI = confidence interval, SD = standard deviation, WHR = waist hip ratio

a) The observational analysis regresses the outcome directly on the exposures BMI and WHR simultaneously. The Poisson regression is adjusted for sex (categorical), age at study entry, alcohol frequency (categorical, from on a daily basis to never), employment (categorical), qualifications (categorical), Townsend deprivation score (categorical in quintiles, where 1 is not deprived and 5 is very deprived), and days of exercise per week (categorical, from 1 to 7).

b) Estimates (with corresponding 95% CIs) represent the fold increase in yearly hospital admission rate per BMI SD (4.74 kg/m^2^) and per WHR SD (0.090). Estimates per BMI and WHR unit are given in Table S3

Observational analyses regressing the outcome on both exposures simultaneously resulted in attenuated associations, with a 1·03-fold increase per BMI SD (95% CI: 1·02, 1·04) and a 1·14-fold increase (95% CI: 1·12, 1·16) per WHR SD (Table 5).

**Table 5:** Association between weighted GRSs for BMI, WHR and WHRadjBMI with BMI and WHR in UK Biobank participants of White British ancestry (*N* = 310,471). Effect estimates are provided per unit (BMI) and per 0.1 unit (WHR).

	**Effect estimate (95% CI)**^**a**^	***P*****-value**	**R**^**2**^**%**	***F***
*BMI GRS (76 SNPs)*				
BMI (unit)	0.112 (0.109 – 0.115)	< 5 × 10^-324^	1.69	5326
WHR (0.1 unit)	0.0075 (0.0069 – 0.0081)	< 4.04 × 10^-144^	0.210	654.1
*WHR GRS (39 SNPs)*				
BMI (unit)	0.0297 (0.0256–0.0337)	< 2.33 × 10^-46^	0.066	204.4
WHR (0.1 unit)	0.0140 (0.0132 – 0.0148)	< 6.40 × 10^-277^	0.406	1267
*WHRadjBMI GRS (48 SNPs)*				
BMI (unit)	-0.024 (−0.0278 to −0.0202)	< 2.41 × 10^-35^	0.050	154
WHR (0.1 unit)	0.0141 (0.0134 – 0.0148)	< 1.88 × 10^-322^	0.474	1477

BMI = body mass index, CI = confidence interval, GRS = genetic risk score, SD = standard deviation, WHR = waist hip ratio, WHRadjBMI = waist hip ratio adjusted for BMI

a) Effect estimate, and corresponding *P*-value represent the change in BMI in units (kg/m^2^) and the change in WHR in 0.1 units per BMI increasing allele (BMI GRS) and WHR increasing allele (WHR GRS, WHRadjBMI GRS). Effect estimates per BMI and WHR SD are given in Table S4.

b) R^2^ values are given in % (e.g., 1.69 here indicates an R^2^ of 0.0169)

For all exposures, estimates derived from adjusted models were lower than those from unadjusted models, but with overlapping confidence intervals.

### 4.3 Association between GRS and exposures

Table 5 shows the associations between BMI and WHR and the BMI GRS, the WHR GRS, and the WHRadjBMI GRS, comprising 76, 39 and 48 SNPs, respectively. In UK Biobank participants of White British ethnicity, each unit increase in BMI GRS was associated with a 0.11 kg/m^2^ higher BMI (95% CI: 0.11, 0.12), with the GRS explaining 1·69% of the variance. Each unit increase in WHR GRS was associated with a 0.01 higher WHR on the 0.10 unit scale (95% CI: 0.01, 0.01), explaining 0.41% of the variance, while each unit increase in WHRadjBMI GRS was associated with a 0.01 increase in WHR on the 0.10 unit scale (95% CI: 0.01, 0.02), explaining 0.47% of the variance. *F*-statistics indicated that each GRS was a strong instrument for Mendelian randomization analyses (Table 5), with all *F*-statistics > 1267.

### 4.4 One-sample Mendelian randomization analyses

One-sample MR estimates of the effect of BMI, WHR and WHRadjBMI on hospital admission rates were obtained per exposure SD. The IV regressions (Table 6), adjusted for age, sex and the first 40 genetic PCAs, yielded a 1·13-fold increase per BMI SD (95% CI: 1·02, 1·27) and a 1·26-fold increase per WHR SD (95% CI: 1·00, 1·58). Using the WHRadjBMI SNPs yielded a 1·22-fold increase per WHR SD (95% CI: 1·01, 1·47). Adjusting for BMI in the WHR regression, by using the residuals from a linear regression of WHR on BMI as an exposure, resulted in a reduced effect of 1·16 (95% CI: 0.97, 1·39) per SD. We have included the adjusted observational estimates from Table 3 for comparison.

**Table 6:** One-sample MR analyses of the effect of BMI (76 SNPs), WHR (39 SNPs) and WHRadjBMI (48 SNPs) on yearly hospital admission rate per year in UK Biobank participants of White British ancestry (*N* = 310,471). Rates and 95% confidence intervals (95% CI) are given. Estimates are provided per exposure SD (SD_BMI_=4.74 and SD_WHR_=0.090).

	**Observational (adjusted**^**a**^**)**	**IV (adjusted**^**b**^**)**
	Rate^d^ 95%CI	Rate^d^ 95%CI
BMI (SD)	1.077 (1.065–1.091)	1.134 (1.015–1.267)
WHR (SD)	1.162 (1.144–1.182)	1.255 (0.997–1.580)
WHRadjBMI (SD)	1.141 (1.120–1.163)	1.216 (1.009–1.466)
WHR~BMI residuals (WHR SD)^c^	1.123 (1.102–1.145)	1.161 (0.968–1.391)

BMI = body mass index, CI = confidence interval, IV = instrumental variable, SD = standard deviation, WHR = waist hip ratio, WHRadjBMI = waist hip ratio adjusted for BMI

a) Adjusted for sex (categorical), age at study entry, alcohol frequency (categorical, from on a daily basis to never), employment (categorical), qualifications (categorical), Townsend deprivation score (categorical in quintiles, where 1 is not deprived and 5 is very deprived), and days of exercise per week (categorical, from 1 to 7). The WHRadjBMI observational analyses also include BMI as a predictor.

b) Adjusted for sex, age at study entry, and 40 PCAs

c) Residuals from linear WHR on BMI regressions are used as an exposure with the WHRadjBMI SNPs as instruments

d) Estimates (with corresponding 95% CIs) represent the fold increase in yearly hospital admission rate per BMI SD (4.74 kg/m^2^) and per WHR SD (0.090). Estimates per BMI and WHR unit are given in Table S5.

Additionally, we examined the effect of BMI, WHR and WHRadjBMI on hospital admission rates across sex (Table 7). While the observational multivariable estimates were comparable for both sexes, the IV analyses resulted in noticeably higher point estimates for the male subset of the data compared to the female one, but with overlapping 95% confidence intervals. For both, the confidence intervals were markedly wider than those in the full data. For the female subset of the data (*N* = 166,610), we observed fold increases of 1·11 (95% CI: 0.95,1·29) per BMI SD and 1·03 (95% CI: 0.86, 1·24) per WHR SD, and a fold increase of 1·06 (95% CI: 0.93, 1·22) per WHR SD, when using the WHRadjBMI SNPs. For the male subset (*N* = 143,861), we observed higher fold increases of 1·16 (95% CI: 1·00, 1·35) per BMI SD, 1·60a (95% CI: 1·11, 2·31) per WHR SD, and, for WHRadjBMI, 1·50 (95% CI: 1·04, 2·17) per WHR SD.

**Table 7:** One-sample MR analyses of the effect of BMI (76 SNPs), WHR (39 SNPs) WHRadjBMI (48 SNPs) on yearly hospital admission rate per year in UK Biobank participants of White British ancestry (*N* = 310,471), per sex (female, *N* = 166,610; male, *N* = 143,861). Rates and 95% confidence intervals (95% CI) are given. Estimates are provided per exposure SD, with in the female subset SD_BMI_= 5.12 and SD_WHR_= 0.070, and in the male subset SD_BMI_= 4.22 and SD_WHR_= 0.065.

	**Female**		**Male**	
	**Observational (adjusted**^**a**^**)**	**IV (adjusted**^**b**^**)**	**Observational (adjusted**^**a**^**)**	**IV (adjusted**^**b**^**)**
	Rate^c^ 95%CI	Rate^c^ 95%CI	Rate^c^ 95%CI	Rate^c^ 95%CI
BMI (SD)	1.078 (1.061–1.095)	1.108 (0.951–1.292)	1.076 (1.057–1.108)	1.162 (0.999–1.352)
WHR (SD)	1.112 (1.094–1.13)	1.032 (0.859–1.241)	1.128 (1.108–1.150)	1.597 (1.105–2.309)
WHRadjBMI (SD)	1.094 (1.074–1.113)	1.062 (0.925–1.219)	1.122 (1.096–1.148)	1.499 (1.035–2.173)

BMI = body mass index, CI = confidence interval, IV = instrumental variable, SD = standard deviation, WHR =waist hip ratio, WHRadjBMI = waist hip ratio adjusted for BMI

a) Adjusted for age at study entry, alcohol frequency (categorical, from on a daily basis to never), employment (categorical), qualifications (categorical), Townsend deprivation score (categorical in quintiles, where 1 is not deprived and 5 is very deprived), and days of exercise per week (categorical, from 1 to 7);

b) Adjusted for age at study entry and 40 PCAs

c) Estimates (with corresponding 95% CIs) represent the fold increase in yearly hospital admission rate per BMI SD (5.12 kg/m^2^ and 4.22 kg/m^2^, for female and male, respectively) and per WHR SD (0.070 and 0.065, for female and male, respectively). Estimates per BMI and WHR unit are given in Table S6.

The multivariable one-sample MR analysis (Table 8) showed no strong evidence for an independent effect of BMI on hospital admissions in the full data, with a fold increase of 1·04 (95% CI: 0.93, 1·15) per BMI SD, when controlling for WHR. Again, we included the adjusted observational effect estimates (from Table 4) for comparison. Conversely, there was evidence for an independent effect of WHR on hospital admissions, with a fold increase of 1·31 (95% CI: 1·04, 1·67) per WHR SD. Instrument strength, as assessed using the conditional Sanderson-Windmeijer *F*-statistic (Sanderson et al., 2019), was sufficient for the multivariable Mendelian randomization analysis, with *F*-statistics of 38·50 and 22·89 for BMI and WHR, respectively.

**Table 8:** Observational multivariable and one-sample multivariable MR analyses of the effect of BMI and WHR on yearly hospital admission rate in UK Biobank participants of White British ancestry (*N* = 310,471). Rates and 95% confidence intervals (95% CI) are given. Estimates are provided per exposure SD (SD_BMI_=4.74 and SD_WHR_=0.090).

	**Observational (adjusted**^**a**^**)**	**IV (adjusted**^**b**^**)**
	Rate^c^ 95%CI	Rate^c^ 95%CI
BMI (per SD)	1.029 (1.015–1.043)	1.035 (0.930–1.153)
WHR (per SD)	1.141 (1.120–1.163)	1.314 (1.037–1.665)

BMI = body mass index, CI = confidence interval, IV = instrumental variable, SD = standard deviation, WHR = waist hip ratio

a) The observational analysis regresses the outcome directly on the exposures BMI and WHR simultaneously. The quasi-Poisson regression is adjusted for sex (categorical), age at study entry, alcohol frequency (categorical, from on a daily basis to never), employment (categorical), qualifications (categorical), Townsend deprivation score (categorical in quintiles, where 1 is not deprived and 5 is very deprived), and days of exercise per week (categorical, from 1 to 7);

b) Adjusted for sex, age at study entry, and 40 PCAs

c) Estimates (with corresponding 95% CIs) represent the fold increase in yearly hospital admission rate per BMI SD (4.74 kg/m^2^) and per WHR SD (0.090). Estimates per BMI and WHR unit are given in Table S7.

### 4.5 Two-sample Mendelian randomization analyses

The IVW estimator showed evidence for a causal effect of all three exposures on hospital admissions (Table 9), at magnitudes consistent with the just-identified one-sample MR results.

**Table 9:** Two-sample MR analysis of hospital admission rate per year in UK Biobank participants of White British ancestry. Rates and 95% confidence intervals (95% CI) are given. Effect estimates are provided per BMI SD (SD_BMI_=4.6) and per WHR SD (SD_WHR_=0.07), for MR-Egger (random effects), IVW (random effects, exact weights), weighted median and weighted mode analyses of BMI (64 SNPs), WHR (34 SNPs) and WHRadjBMI (45 SNPs). SNPs with an LD R2 < 0.001 have been retained.

			MR-Egger			
		IVW (random effects, exact weights)	Intercept	Slope	Penalized weighted median	Weighted mode
BMI (SD)	Rate^a^ (95% CI)	1.098	1.004	0.973	1.095	1.095
		(1.009–1.194)	(0.997–1.011)	(0.759–1.247)	(0.930–1.290)	(0.915–1.312)
WHR (SD)	Rate^a^ (95% CI)	1.199	1.000	1.185	1.236	1.204
		(1.054–1.364)	(0.987–1.014)	(0.676–2.079)	(0.985–1.550)	(0.867–1.167)
WHRadjBMI (SD)	Rate^a^ (95% CI)	1.151	1.008	0.870	1.124	1.076
		(1.028–1.287)	(0.995–1.021)	(0.541–1.400)	(0.941–1.343)	(0.819–1.414)

BMI = body mass index, CI = confidence interval, IVW = inverse variance weighted, MR = Mendelian randomization, SD = standard deviation, WHR = waist hip ratio, WHRadjBMI = waist hip ratio adjusted for BMI.

a) Adjusted for sex, age and the first 40 genetic principal components. Estimates (with corresponding 95% CIs) represent the fold increase in yearly hospital admission rate per BMI SD (4.6 kg/m2) and per WHR SD (0.07). Estimates per BMI and WHR unit are given in Table S8.

For BMI, we observed a fold increase of 1·10 (95% CI: 1·01, 1·19) per SD; for WHR, a fold increase of 1·20 (95% CI: 1·05, 1·36) per SD; and for WHRadjBMI, a fold increase of 1·15 (95% CI: 1·03, 1·29) per SD. The penalized weighted median and weighted mode yielded near identical point estimates, albeit with wider confidence intervals that included the null.

The widest confidence intervals were observed for the MR-Egger estimates, as is usually the case given the lower power of this estimator, since it estimates twice the number of parameters (both the intercept and the slope coefficient) compared to the other estimators. While for WHR, the MR-Egger point estimate was comparable to the previous estimates, it was lower for BMI and WHRadjBMI. The MR-Egger intercept indicated no directional pleiotropy.

Heterogeneity was assessed using Rücker’s Q' (Q_R_) and Cochran’s Q (Q_C_). For all three exposures, the Q-statistics were smaller than the number of SNPs used for estimation (Q_R,BMI_=51.29, Q_C,BMI_=52.06; Q_R,WHR_=28.64, Q_C,WHR_=28.92; Q_R,WHRadj_=43.80, Q_C,WHRadj_=44.01). Despite the lack of substantial heterogeneity, we performed a sensitivity analysis to investigate the potential presence of pleiotropy driven by outlying SNPs. Individual SNPs were identified in a visual inspection of leave-one-out plots (Figs. S1-S3) and removed, with 3, 4 and 4 SNPs excluded for BMI, WHR, and WHRadjBMI, respectively. The two-sample MR analyses were repeated using the reduced set of SNPs, yielding comparable point estimates and confidence intervals overlapping with those from the original analyses (Table S9).

A multivariable two-sample IVW analysis of BMI and WHR simultaneously (Table 10) yielded no strong evidence for an association of BMI and the yearly hospital admission rate, with a fold increase of 0.99 per BMI SD (95% CI: 0.85, 1·14), but indicated a strong association between WHR and hospital admission rate, with a fold increase of 1·30 per WHR SD (95% CI: 1·02, 1·65).

**Table 10:** Multivariable two-sample MR IVW estimates for the effect of BMI and WHR (70 SNPs) on yearly hospital admission rate in UK Biobank participants of White British ancestry. Rates and 95% confidence intervals (95% CI) are given. Effect estimates are provided per BMI SD (SD_BMI_=4.6) and per WHR SD (SD_WHR_=0.07). SNPs with an LD R^2^ < 0.001 were retained.

	**Rate^a^**	**95% CI**
BMI (SD)	0.986	(0.850–1.143)
WHR (SD)	1.297	(1.022–1.647)

BMI = body mass index, CI = confidence interval, IVW = inverse variance weighted, MR = Mendelian randomization, SD = standard deviation, WHR = waist hip ratio

a) Adjusted for sex, age and the first 40 genetic principal components. Estimates (with corresponding 95% CIs) represent the fold increase in yearly hospital admission rate per BMI SD (4.6 kg/m^2^) and per WHR SD (0.07). Estimates per BMI and WHR unit are given in Table S10.

The gene-exposure associations and gene-outcome associations for the univariable two-sample MR analyses are given in Tables S11, S12 and S13 for BMI, WHR, and WHRadjBMI, respectively. The same quantities are provided for the multivariable two-sample analysis in Table S14.

### 4.6 Comparison across methods

**Fig. 3:**
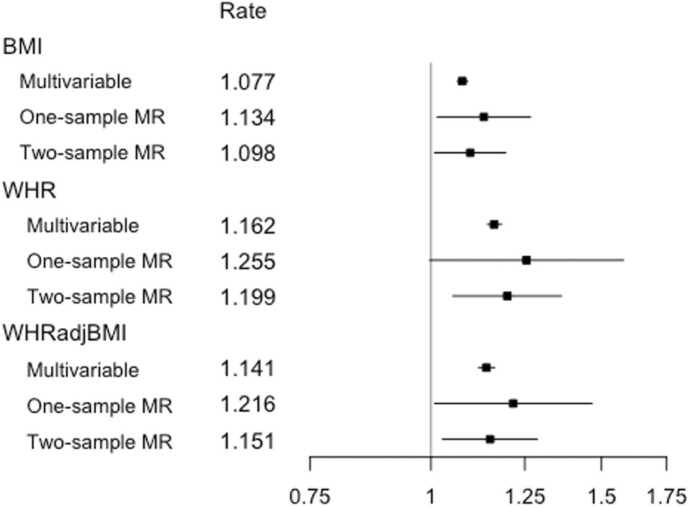
Estimates from multivariable observational analyses, one-sample MR analyses and two-sample MR IVW analyses for exposures BMI, WHR and WHRadjBMI per SD unit. Shown are point estimates, alongside 95% CIs, for the effect of each exposure on yearly hospital admission rate in UK Biobank participants of White British ancestry. The results are plotted on the log scale, to ensure symmetrical CIs and comparability of magnitude across estimates. Rate estimates and x-axis values are given on the rate scale. All Mendelian randomization analyses were adjusted for age, sex and the first 40 genetic PCAs. The multivariable observational analyses were adjusted for a range of baseline patient characteristics (Table 1). Notes: BMI = body mass index, CI = confidence interval, IVW = inverse variance weighted, MR = Mendelian randomization, SD = standard deviation, WHR = waist hip ratio, WHRadjBMI = waist hip ratio adjusted for BMI.

Fig. 3 summarizes the effect of each exposure on yearly hospital admission rate, as estimated from traditional multivariable analyses, and one- and two-sample MR analyses.

For all exposures, we observed a higher point estimate for the one-sample MR analyses when compared to the traditional analyses. On the whole, the estimated effects were consistent across estimators, with overlapping confidence intervals, and comparatively wider confidence intervals for WHR and WHRadjBMI.

**Fig. 4:**
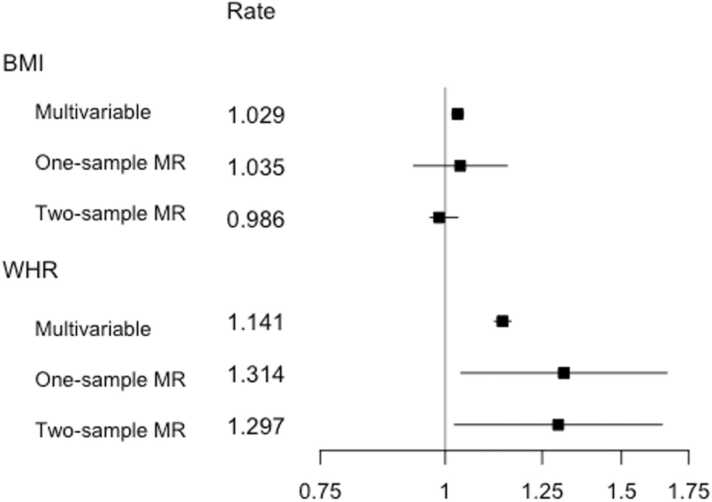
Estimates from multivariable observational analyses, multivariable one-sample MR analyses and multivariable two-sample MR IVW analyses for exposures BMI and WHR per SD unit. Shown are point, estimates, alongside 95% CIs for the effect of each exposure on yearly hospital admission rate in UK Biobank participants of White British ancestry. The results are plotted on the log scale, to ensure symmetrical CIs and comparability of magnitude across estimates. Rate estimates and x-axis values are given on the rate scale. MR analyses were adjusted for age, sex and the first 40 genetic PCAs. The multivariable observational analyses were adjusted for a range of baseline patient characteristics (Table 1), with the BMI estimate adjusted for WHR and vice versa. Notes: BMI = body mass index, CI = confidence interval, IVW = inverse variance weighted, MR = Mendelian randomization, SD = standard deviation, WHR = waist hip ratio.

A similar pattern is observed in Fig. 4, which summarizes the multivariable estimator results. Once more, the confidence intervals for both exposures overlapped, but with the BMI point estimates more closely grouped together. In contrast to the univariable analyses summarized in Fig. 3, the one- and two-sample BMI estimate confidence intervals now included the null.

## 5 Discussion

We report the first Mendelian randomization (MR) analysis of all-cause hospital admissions in relation to adiposity. Our MR results, based on analysis of more than 550,000 hospital admissions measured in over 300,000 UK Biobank participants, identified a causal effect of a higher BMI, WHR and WHRadjBMI on increased hospital admission risk, using one- and two-sample MR methods, in both a univariable and multivariable MR framework.

In the multivariable one-sample and two-sample MR analyses, a relatively stronger positive effect was observed for WHR than for BMI, suggesting that the relationship between adiposity and hospital admissions may be driven by a detrimental distribution of fat and adipose tissue rather than by BMI itself. WHR has been little investigated previously in the context of hospital admissions, and our results emphasize the relevance of considering WHR as a measure of adiposity in addition to BMI in this context. Results from sensitivity analyses that relaxed the exclusion restriction were broadly concordant across methods relying on different assumptions, suggesting that the same causal effect was being identified.

A positive association of BMI and all-cause hospital admissions was reported in observational studies investigating populations from the UK (Kent et al., 2017b, O'Halloran et al., 2020), Australia (Korda et al., 2015), Canada (Chen et al., 2007), Italy (Migliore et al., 2013), and the USA (Buys et al., 2014, Han et al., 2009). An observational study of approximately 1·09 million UK women found a yearly hospital admission rate increase of 1·12 (95% CI: 1·12, 1·13) for every 5 kg/m^2^ increase in BMI (Reeves et al., 2014). In just over 300,000 individuals, we observed estimates amounting to rate coefficients of 1·02^5^ = 1·08 (95% CI: 1·07, 1·10), 1·14 (95% CI: 1·02, 1·28) and 1·10 (95% CI: 1·01, 1·21) from the multivariable observational, one-sample MR and two-sample MR analyses, respectively, giving effect estimates of a magnitude comparable to those observed previously in UK women from observational studies.

Another observational study, examining the association between BMI and hospital admissions in 451,320 UK Biobank participants, found increases in yearly hospital admission rates, measured per 2 kg/m^2^ BMI, of 1·06 (95% CI: 1·05, 1·07) and 1·06 (95% CI: 1·05, 1·07) for male and female never-smokers, respectively (O'Halloran, 2020). We observed comparable estimates, with increases across both sexes combined, per 2 kg/m^2^ BMI, of 1·02^2^ = 1·03 (95% CI: 1·03, 1·04), 1·05 (95% CI: 1·01, 1·11), and 1·04 (95% CI: 1·00, 1·08), for the multivariable observational, one-sample MR and two-sample MR analyses, respectively. For the sex-specific analyses, the estimates were also comparable, with increases per 2 kg/m^2^ BMI of 1·03 (95% CI: 1·02, 1·04) for the multivariable observational analyses of both the female and male subsets, and increases of 1·04 (95% CI: 0.98, 1·10) and 1·07 (95% CI: 1·00, 1·16) for the one-sample MR analyses of the female and male subsets, respectively

While the effect of BMI/WHR on hospital admission rate has not previously been studied in this context, the effect of BMI on hospital cost was examined in a two-sample MR analysis, using data from UK Biobank, which largely overlaps with our own study population (Dixon et al., 2020). A positive causal effect of BMI on hospital cost was found in that study, in line with our own observation of increased hospital admissions for higher BMI.

### 5.1 Limitations

MR methods make it possible to avoid certain biases common to traditional epidemiological studies, but also face limitations, both in terms of interpretation and in terms of potential alternative sources of bias. When interpreting the results, it should be noted that MR does not estimate an average treatment effect, but rather a local average treatment effect (LATE) instead, under the assumption that the effect of IV on treatment is, for all IVs, in the same direction for all subjects – the condition of monotonicity (Von Hinke et al., 2016). This means that we estimated the effect of WHR and BMI in those subjects whose WHR/BMI exposure values differ on varying the levels of the respective IVs, under the condition that this change occurs in the same direction for all participants. This is probably a reasonable assumption.

As the IVs are comprised of genetic markers, which are “assigned” at conception, the estimated LATE is a measure of the effects of a lifelong exposure to BMI-increasing alleles/WHR-increasing alleles. Additionally, we should note that, for all three exposures, a relatively modest percentage of variance is explained by the genetic variants, reducing statistical power for detecting the effect of a change in BMI/WHR and consequently resulting in less precise (Haycock et al., 2016) (albeit potentially less biased) estimates.

A key assumption of MR is that of gene-environment equivalence: that genetically influenced BMI and WHR will have the same effect on hospital admission risk as, for example, adiposity modified by diet and/or exercise. The included SNPs, however, may not meet the stable unit treatment assumption (SUTVA) (Burgess and Thompson, 2015) and, therefore, our estimates of the effect of BMI and WHR will not necessarily be representative of the increase or reduction in hospital admission rate when adiposity is altered through interventions.

We were limited to inpatient hospital admissions as the source of hospital data linked to UK Biobank at the time of writing. It is possible that other forms of hospital and primary care may substitute for inpatient care, in which case our estimates may overstate the effect of adiposity on overall healthcare admissions and care episodes. On the other hand, if inpatient care is complementary to other forms of care, then we may have understated effect sizes. In practice, both influences may be present. Our results are best interpreted in relation to our outcome of inpatient admissions.

While UK Biobank is a unique and high-quality source, the underlying demographic structure of the data imposes various restrictions in terms of generalizability. The UK Biobank sample is healthier and wealthier than the population from which it is drawn and consequently is likely to not be representative of the wider UK population. Indeed, we observed lower rates of mortality than in the general population (Bycroft et al., 2018), better health-related behavior and a higher level of education (Fry et al., 2017).

As less healthy individuals are less likely to participate in the study, the observed sample may be subject to selection bias (Glymour, 2006, Munafò et al., 2018, Spirtes et al., 1993), which will impact the estimates obtained from both conventional multivariable analyses and MR analyses – where estimates obtained may even be an underestimation of the effect of adiposity on hospital admissions in the general population. Within the observed sample, 2% of participants died before the end of follow-up. It is plausible these individuals were in poorer health and had on average worse outcomes (more frequent hospital admissions) than those observed for the full length of study, which may also contribute to the underestimation of the effect, resulting in a conservative estimate of adiposity on hospital admissions. It should also be noted that current analyses have been limited to individuals of White British ancestry, and, consequently, that the results will not necessarily generalize to other ancestral groups.

A potential further limitation is the possibility of cohort effects. UK Biobank participants are aged 39–72 years, giving rise to a range of birth cohorts in our data sample. There is some evidence from other sources to suggest that SNPs exert a greater influence on BMI for those born in more recent decades, possibly because of an increasingly obesogenic environment (Hartwig et al., 2018, Walter et al., 2016). In this case, other things being equal, our estimates may underestimate the impact of adiposity on hospital admissions.

Additional sources of bias that should be considered in the context of MR and the UK Biobank data are assortative mating (Hartwig et al., 2018, Jacobson et al., 2007, Morris et al., 2019, Tenesa et al., 2016), confounding due to population stratification bias (Brumpton et al., 2020, Haworth et al., 2019, Koellinger and de Vlaming, 2019, Morris et al., 2019) and the presence of dynastic effects (Brumpton et al., 2020, Fletcher, 2011, Morris et al., 2019), which have all been implicated with respect to BMI previously. While we attempted to minimize the impact of population stratification with the inclusion of 40 genetic principal components, latent data structure may still exist for some exposures such as BMI (Haworth et al., 2019). Within-family analysis may address some of these issues (Brumpton et al., 2020, Davies et al., 2019), although statistical power for this form of modeling is limited given the relatively modest available sample sizes of related individuals in UK Biobank (Howe et al., 2020).

## 6 Conclusion

This study describes the first Mendelian randomization analysis to estimate the causal effect of BMI, WHR and WHRadjBMI on yearly hospital admission rates. Results supported the causal role of greater adiposity in increasing the risk of hospital admissions. Causal point estimates were larger than those obtained from conventional observational models, further emphasizing the necessity of exploring policies intended to address adverse adiposity profiles.

Multivariable Mendelian randomization analyses suggested that the effect of BMI on hospital admission rates may be mediated by WHR, and that an unfavorable fat distribution may drive the relationship between increased adiposity and higher hospital admission rates. Additionally, we demonstrate that a non-standard outcome like hospital admission counts can be successfully modeled using Mendelian randomization methods, both in a one-sample and two-sample framework, by replacing the second stage regression (modeling the gene-outcome association) with a Poisson regression.

## Declarations

### Funding statement

AH, RCR, KHW and PD are members of the MRC Integrative Epidemiology Unit at the University of Bristol, which is supported by the 10.13039/501100000265Medical Research Council and the 10.13039/501100000883University of Bristol (MC_UU_00011/1, MC_UU_00011/3, MC_UU_00011/9). PD acknowledges support from a Medical Research Council Skills Development Fellowship (Mendelian randomization/P014259/1). RCR is a de Pass Vice Chancellor’s Research Fellow at the University of Bristol. KHW was supported by the Elizabeth Blackwell Institute for Health Research, University of Bristol and the Wellcome Trust Institutional Strategic Support Fund (204813/Z/16/Z) and works within a group funded by the Wellcome Trust Investigator Award (202802/Z/16/Z).

## Role of the funding source

The funding source had no role in study design, data collection, data analysis, data interpretation or writing of the report. All authors had full access to all the data in the study and accept responsibility for the decision to submit for publication.

## CRediT authorship contribution statement

**Audinga-Dea Hazewinkel:** Writing − original draft preparation, Visualization, Formal analysis, Software. **Kaitlin Wade:** Writing − review & editing. **Rebecca Richmond:** Conceptualization, Writing − review & editing. **Padraig Dixon:** Conceptualization, Supervision, Data curation, Writing − review & editing.

## Declaration of interest statement

The authors declare no conflicts of interest.
