# Supplementary material for: Mendelian randomization analysis of the causal impact of body mass index and waist-hip ratio on rates of hospital admission
Source: Econ Hum Biol. 2022 Jan;44:101088. doi: 10.1016/j.ehb.2021.101088 (PMC8784824; doi:10.1016/j.ehb.2021.101088)

# Appendix

**Supplementary tables and figures**

*Tables*

**Table S1.** Cross-table of participant counts across eight exclusion criteria. 1

**Table S2.** Observational multivariable analysis of the effect of BMI, WHR and WHR 2

adjusted for BMI on yearly hospital admission rate

**Table S3.** Observational multivariable analysis of the effect of BMI and WHR jointly on 2

yearly hospital admission rate

**Table S4.** Association between weighted GRS for BMI, WHR and WHRadjBMI with BMI 3

and WHR

**Table S5.** One-sample MR analysis of the effect of BMI, WHR and WHRadjBMI on yearly 4

hospital admission rate

**Table S6.** One-sample MR analysis of the effect of BMI, WHR and WHRadjBMI on yearly 4

hospital admission rate per sex

**Table S7.** Observational multivariable and one-sample multivariable MR analyses of the 5

effect of BMI and WHR on yearly hospital admission rate

**Table S8.** Two-sample MR analyses; MR-Egger, IVW, weighted median and weighted mode 6

analyses of BMI, WHR and WHRadjBMI on yearly hospital admission rate

**Table S9.** Two-sample MR analyses; MR-Egger, IVW, weighted median and weighted mode 6

analyses of BMI, WHR and WHRadjBMI on yearly hospital admission rate:

a sensitivity analysis on exclusion of potentially pleiotropic SNPs

**Table S10.** Multivariable two-sample MR IVW estimates of the effect of BMI and WHR 7

jointly on yearly hospital admission rate

**Table S11.** BMI-SNP and hospital admission count-SNP associations 8

**Table S12.** WHR-SNP and hospital admission count-SNP associations 10

**Table S13.** WHRadjBMI-SNP and hospital admission count-SNP associations 12

**Table S14.** BMI-SNP, WHR-SNP and hospital admission count-SNP associations as used 14

for the multivariable two-sample MR analysis.

*Figures*

**Figure S1.** Plots for two-sample MR analysis of BMI effect on yearly hospital admission 16

rate: A) Cochran’s Q against Rücker’s Q; B) SNP effects on log scale

**Figure S2.** Plots for two-sample MR analysis of WHR effect on yearly hospital admission 17

rate: A) Cochran’s Q against Rücker’s Q; B) SNP effects on log scale

**Figure S3.** Plots for two-sample MR analysis of WHRadjBMI effect on yearly hospital 18

admission rate: A) Cochran’s Q against Rücker’s Q; B) SNP effects on log scale

| **Table S1.** Cross-table of participant count across eight exclusion criteria. Total participant count per exclusion criteria is given on the diagonal, with the remaining table entries giving the number of participants meeting any two such criteria (e.g. 345 participants failed the standard inclusion criteria and were of non-white British ancestry). A total of 154901 participants match one or more exclusion criteria and are considered ineligible for analysis | | | | | | | | |
| --- | --- | --- | --- | --- | --- | --- | --- | --- |
| *Exclusion criterion* | Incorrect admission information^a^ | Failed standard inclusion criteria^b^ | Non-white British ancestry | Highly related^c^ | Minimally related^d^ | Not genotyped^e^ | No BMI/WHR measurements | No PCA information |
| Incorrect admission information^a^ | **34** | 0 | 1 | 0 | 1 | 27 | 1 | 27 |
| Failed  standard inclusion criteria^b^ |  | **1675** | 345 | 0 | 100 | 899 | 8 | 0 |
| Non-white British  ancestry |  |  | **73278** | 7 | 7431 | 225 | 415 | 0 |
| Highly  Related^c^ |  |  |  | **8** | 0 | 0 | 0 | 0 |
| Minimally related^d^ |  |  |  |  | **73893** | 0 | 255 | 0 |
| Not  Genotyped^e^ |  |  |  |  |  | **13797** | 796 | 12898 |
| No BMI/WHR measurements |  |  |  |  |  |  | **2454** | 794 |
| No PCA information |  |  |  |  |  |  |  | 12899 |
| a) admissions prior to study start date, post death/censoring date or registered death prior to study start; b) individuals that have a mismatch between genetically inferred and reported gender, individuals with sex chromosome types putatively different from XX or XY and individuals that are outliers in heterozygosity and missing rate; c) individuals related to more than 200 other participants; d) on exclusion a maximal set of unrelated individuals is retained; ) not genotyped for the exposures of interest (BMI, WHR, WHRadjBMI) | | | | | | | | |

| **Table S2.** Observational multivariable analysis of the effect of BMI, WHR and WHR adjusted for BMI on yearly hospital admission rate per year in UK Biobank participants of White British ancestry (*N*=310,471). Rates and 95% confidence intervals (95% CI) are given. Effect estimates are provided per unit (BMI), per 0.1 unit (WHR) and per SD (SD_BMI_=4.74 and SD_WHR_=0.090). |
| --- |

|  | **Observational (unadjusted)** | **Observational (adjusted^a^)** |
| --- | --- | --- |
|  | Rate^b^ 95%CI | Rate^b^ 95%CI |
| BMI (unit) | 1.030 (1.027-1.032) | 1.016 (1.013-1.018) |
| BMI (SD) | 1.148 (1.133-1.163) | 1.077 (1.065-1.091) |
| WHR (0.1 unit) | 1.221 (1.203-1.240) | 1.182 (1.160-1.203) |
| WHR (SD) | 1.197 (1.181-1.214) | 1.162 (1.144-1.182) |
| WHRadjBMI (0.1 unit) | 1.173 (1.153-1.194) | 1.157 (1.133-1.182) |
| WHRadjBMI (SD) | 1.155 (1.137-1.173) | 1.141 (1.120-1.163) |
| WHR~BMI residuals  (0.1 WHR unit)^c^ | 1.174 (1.154-1.194) | 1.138 (1.114-1.162) |
| WHR~BMI residuals (WHR SD)^c^ | 1.156 (1.136-1.176) | 1.123 (1.102-1.145) |
| **NOTES TO TABLE:** BMI = body mass index, CI = confidence interval, SD = standard deviation, WHR = waist-hip-ratio, WHRadjBMI = waist-hip-ratio adjusted for BMI  a) Adjusted for sex (categorical), age at study entry, alcohol frequency (categorical, from on a daily basis to never), employment (categorical), qualifications (categorical), Townsend deprivation score (categorical in quintiles, where 1 is not deprived and 5 is very deprived), and days of exercise per week (categorical, from 1 to 7). The WHRadjBMI observational analyses also include BMI as a predictor.  b) Estimates (with corresponding 95% CIs) represent the fold increase in yearly hospital admission rate per BMI unit (1 kg/m^2^) and SD (4.74 kg/m^2^) and per 0.1 WHR unit and SD (0.090) | | |

| **Table S3.** Observational multivariable analysis of the effect of BMI and WHR jointly on yearly hospital admission rate in UK Biobank participants of White British ancestry (*N*=310471). Rates and 95% confidence intervals (95% CI) are given. Estimates are provided per unit (BMI), per 0.1 unit (WHR) and per exposure SD (SD_BMI_=4.74 and SD_WHR_=0.090). | | |
| --- | --- | --- |
|  | | |
|  | **Observational (unadjusted)** | **Observational (adjusted^a^)** |
|  | Rate^b^ 95% CI | Rate^b^ 95% CI |
| BMI (per unit) | 1.017 (1.014-1.020) | 1.006 (1.003-1.009) |
| BMI (per SD) | 1.084 (1.068-1.100) | 1.029 (1.015-1.043) |
| WHR (per 0.1 unit) | 1.173 (1.153-1.194) | 1.158 (1.134-1.182) |
| WHR (per SD) | 1.155 (1.137-1.173) | 1.141 (1.120-1.163) |
| **NOTES TO TABLE:** BMI = body mass index, CI = confidence interval, SD = standard deviation, WHR = waist-hip-ratio  a) The observational analysis regresses the outcome directly on the exposures BMI and WHR simultaneously. The Poisson regression is adjusted for sex (categorical), age at study entry, alcohol frequency (categorical, from on a daily basis to never), employment (categorical), qualifications (categorical), Townsend deprivation score (categorical in quintiles, where 1 is not deprived and 5 is very deprived), and days of exercise per week (categorical, from 1 to 7);  b) Estimates (with corresponding 95% CIs) represent the fold increase in yearly hospital admission rate per BMI unit (1 kg/m^2^) and SD (4.74 kg/m^2^) and per 0.1 WHR unit and SD (0.090) | | |

| **Table S4**. Association between weighted GRS for BMI, WHR and WHRadjBMI with BMI and WHR in UK Biobank participants of White British ancestry (*N*=310471). Effect estimates are provided per unit (BMI), per 0.1 unit (WHR) and per SD (SD_BMI_=4.74 and SD_WHR_=0.090). | | | | |
| --- | --- | --- | --- | --- |
|  | **Effect estimate (95% CI)^a^** | ***P*-value** | **R^2^ %** | ***F*** |
| *BMI GRS (76 SNPs)* | | | |  |
| BMI (unit) | 0.112 (0.109 – 0.115) | <5 x 10^-324^ | 1.69 | 5326 |
| BMI (SD) | 0.0236 (0.023 – 0.0243) | <5 x 10^-324^ | 1.69 | 5326 |
| WHR (0.1 unit) | 0.0075 (0.0069 – 0.0081) | <4.04 x 10^-144^ | 0.210 | 654.1 |
| WHR (SD) | 0.0084 (0.0077 – 0.009) | <4.04 x 10^-144^ | 0.210 | 654.1 |
|  |  |  |  |  |
| *WHR GRS (39 SNPs)* | | | |  |
| BMI (unit) | 0.0297 (0.0256-0.0337) | <2.33 x 10^-46^ | 0.066 | 204.4 |
| BMI (SD) | 0.0060 (0.005-0.007) | <2.33 x 10^-46^ | 0.066 | 204.4 |
| WHR (0.1 unit) | 0.0140 (0.0132 – 0.0148) | <6.40 x 10^-277^ | 0.406 | 1267 |
| WHR (SD) | 0.0156 (0.0147 – 0.0164) | <6.40 x 10^-277^ | 0.406 | 1267 |
|  |  |  |  |  |
| *WHRadjBMI GRS (48 SNPs)* | | | |  |
| BMI (unit) | -0.024 (-0.0278 – -0.0202) | <2.41 x 10^-35^ | 0.050 | 154 |
| BMI (SD) | -0.005 (-0.006 – -0.004) | <2.41 x 10^-35^ | 0.050 | 154 |
| WHR (0.1 unit) | 0.0141 (0.0134 – 0.0148) | <1.88 x 10^-322^ | 0.474 | 1477 |
| WHR (SD) | 0.0157 (0.0149 – 0.0165) | <1.88 x 10^-322^ | 0.474 | 1477 |
| **NOTES TO TABLE**: BMI = body mass index, CI = confidence interval, GRS = genetic risk score, SD = standard deviation, WHR = waist-hip-ratio, WHRadjBMI = waist-hip-ratio adjusted for BMI  a) Effect estimate, and corresponding *P*-value represent the change in BMI in units (kg/m^2^) and SD units (4.74 kg/m^2^) and the change in WHR in 0.1 units and SD units (0.090) per BMI increasing allele (BMI GRS) and WHR increasing allele (WHR GRS, WHRadjBMI GRS) | | | | |

| **Table S5.** One-sample MR analyses of the effect of BMI (76 SNPs), WHR (39 SNPs) and WHRadjBMI (48 SNPs) on yearly hospital admission rate per year in UK Biobank participants of White British ancestry (*N*=310471). Rates and 95% confidence intervals (95% CI) are given. Estimates are provided per unit (BMI), per 0.1 unit (WHR) and per exposure SD (SD_BMI_=4.74 and SD_WHR_=0.090). | | | |
| --- | --- | --- | --- |
|  | **Observational (adjusted^a^)** | **IV (adjusted^b^)** |  |
|  | Rate^d^ 95%CI | Rate^d^ 95%CI |  |
| BMI (unit) | 1.016 (1.013-1.018) | 1.027 (1.003-1.051) |  |
| BMI (SD) | 1.077 (1.065-1.091) | 1.134 (1.015-1.267) |  |
| WHR (0.1 unit) | 1.182 (1.160-1.203) | 1.287 (0.997-1.661) |  |
| WHR (SD) | 1.162 (1.144-1.182) | 1.255 (0.997-1.580) |  |
| WHRadjBMI (0.1 unit) | 1.157 (1.133-1.182) | 1.242 (1.010-1.529) |  |
| WHRadjBMI (SD) | 1.141 (1.120-1.163) | 1.216 (1.009-1.466) |  |
| WHR~BMI residuals  (0.1 WHR unit)^c^ | 1.138 (1.114-1.162) | 1.180 (0.965-1.442) |  |
| WHR~BMI residuals (WHR SD)^c^ | 1.123 (1.102-1.145) | 1.161 (0.968-1.391) |  |
| **NOTES TO TABLE**: BMI = body mass index, CI = confidence interval, IV = instrumental variable, SD = standard deviation, WHR = waist-hip-ratio, WHRadjBMI = waist-hip-ratio adjusted for BMI  a) Adjusted for sex (categorical), age at study entry, alcohol frequency (categorical, from on a daily basis to never), employment (categorical), qualifications (categorical), Townsend deprivation score (categorical in quintiles, where 1 is not deprived and 5 is very deprived), and days of exercise per week (categorical, from 1 to 7). The WHRadjBMI observational analyses also include BMI as a predictor.  b) Adjusted for sex, age at study entry, and 40 PCAs  c) Residuals from linear WHR on BMI regressions are used as an exposure with the WHRadjBMI SNPs as instruments  d) Estimates (with corresponding 95% CIs) represent the fold increase in yearly hospital admission rate per BMI unit and SD (4.74 kg/m^2^) and per WHR 0.1 unit and SD (0.090) | | | |

| **Table S6.** One-sample MR analyses of the effect of BMI (76 SNPs), WHR (39 SNPs) WHRadjBMI (48 SNPs) on yearly hospital admission rate per year in UK Biobank participants of White British ancestry (*N*=310471), per sex (female, *N*=166610; male, *N*=143861). Rates and 95% confidence intervals (95% CI) are given. Estimates are provided per unit (BMI), per 0.1 unit (WHR) and per exposure SD, with in the female subset SD_BMI_=5.12 and SD_WHR_=0.070, and in the male subset SD_BMI_=4.22 and SD_WHR_=0.065. | | | | |
| --- | --- | --- | --- | --- |
|  | **Female** | | **Male** | |
|  | **Observational (adjusted^a^)** | **IV (adjusted^b^)** | **Observational (adjusted^a^)** | **IV (adjusted^b^)** |
|  | Rate^c^ 95%CI | Rate^c^ 95%CI | Rate^c^ 95%CI | Rate^c^ 95%CI |
| BMI (unit) | 1.015 (1.012-1.018) | 1.020 (0.990-1.051) | 1.017 (1.013-1.022) | 1.036 (1.000-1.074) |
| BMI (SD) | 1.078 (1.061-1.095) | 1.108 (0.951-1.292) | 1.076 (1.057-1.108) | 1.162 (0.999-1.352) |
| WHR (0.1 unit) | 1.164 (1.138-1.191) | 1.046 (0.804-1.362) | 1.204 (1.170-1.239) | 2.056 (1.166-3.623) |
| WHR (SD) | 1.112 (1.094-1.130) | 1.032 (0.859-1.241) | 1.128 (1.108-1.150) | 1.597 (1.105-2.309) |
| WHRadjBMI (0.1 unit) | 1.137 (1.108-1.166) | 1.090 (0.895-1.328) | 1.193 (1.152-1.236) | 1.865 (1.054-3.299) |
| WHRadjBMI (SD) | 1.094 (1.074-1.113) | 1.062 (0.925-1.219) | 1.122 (1.096-1.148) | 1.499 (1.035-2.173) |
| **NOTES TO TABLE**: BMI = body mass index, CI = confidence interval, IV = instrumental variable, SD = standard deviation, WHR = waist-hip-ratio, WHRadjBMI = waist-hip-ratio adjusted for BMI  a) The observational analysis regresses the outcome directly on the exposures BMI and WHR simultaneously. The Poisson regression is adjusted for sex (categorical), age at study entry, alcohol frequency (categorical, from on a daily basis to never), employment (categorical), qualifications (categorical), Townsend deprivation score (categorical in quintiles, where 1 is not deprived and 5 is very deprived), and days of exercise per week (categorical, from 1 to 7);  b) Adjusted for sex, age at study entry, and 40 PCAs  c) Estimates (with corresponding 95% CIs) represent the fold increase in yearly hospital admission rate per BMI unit and SD (5.12 kg/m^2^ and 4.22 kg/m^2^, for female and male, respectively) and per WHR 0.1 unit and SD (0.070 and 0.065, for female and male, respectively) | | | | |

| **Table S7.** Observational multivariable and one-sample multivariable MR analyses of the effect of BMI and WHR on yearly hospital admission rate in UK Biobank participants of White British ancestry (*N*=310471). Rates and 95% confidence intervals (95% CI) are given. Estimates are provided per unit (BMI), per 0.1 unit (WHR) and per exposure SD (SD_BMI_=4.74 and SD_WHR_=0.090). | | | |
| --- | --- | --- | --- |
|  | **Observational (adjusted^a^)** | **IV (adjusted^b^)** |  |
|  | Rate^c^ 95%CI | Rate^c^ 95%CI |  |
| BMI (unit) | 1.006 (1.003-1.009) | 1.007 (0.985-1.031) |  |
| BMI (SD) | 1.029 (1.015-1.043) | 1.035 (0.930-1.153) |  |
| WHR (0.1 unit) | 1.158 (1.134-1.182) | 1.354 (1.041-1.761) |  |
| WHR (SD) | 1.141 (1.120-1.163) | 1.314 (1.037-1.665) |  |
| **NOTES TO TABLE**: BMI = body mass index, CI = confidence interval, IV = instrumental variable, SD = standard deviation, WHR = waist-hip-ratio  a) The observational analysis regresses the outcome directly on the exposures BMI and WHR simultaneously. The Poisson regression is adjusted for sex (categorical), age at study entry, alcohol frequency (categorical, from on a daily basis to never), employment (categorical), qualifications (categorical), Townsend deprivation score (categorical in quintiles, where 1 is not deprived and 5 is very deprived), and days of exercise per week (categorical, from 1 to 7);  b) Adjusted for sex, age at study entry, and 40 PCAs  c) Estimates (with corresponding 95% CIs) represent the fold increase in yearly hospital admission rate per BMI unit (1 kg/m^2^) and SD (4.74 kg/m^2^) and per 0.1 WHR unit and SD (0.090) | | | |

| **Table S8.** Two-sample MR analysis of hospital admission rate per year in UK Biobank. Rates and 95% confidence intervals (95% CI) are given. Effect estimates are provided per BMI SD (SD_BMI_=4.6) and per WHR SD (SD_WHR_=0.07). | | | | | | |
| --- | --- | --- | --- | --- | --- | --- |
|  |  |  | MR-Egger | |  |  |
|  |  | IVW (random effects, exact weights) | Intercept | Slope | Penalized weighted median | Weighted mode |
| BMI (unit) | Rate^a^  (95% CI) | 1.020  (1.002-1.038) | 1.001  (0.999-1.002) | 0.994  (0.943-1.048) | 1.020  (0.986-1.055) | 1.019  (0.981-1.059) |
| BMI (SD) | Rate^a^  (95% CI) | 1.098  (1.009-1.194) | 1.004  (0.997-1.011) | 0.973  (0.759-1.247) | 1.095  (0.930-1.290) | 1.095  (0.915-1.312) |
| WHR (0.1 unit) | Rate^a^   (95% CI) | 1.223  (1.062-1.407) | 1.000  (0.987-1.014) | 1.208  (0.648-2.253) | 1.265  (0.982-1.630) | 1.229  (0.864-1.748) |
| WHR (SD) | Rate^a^   (95% CI) | 1.199  (1.054-1.364) | 1.000  (0.987-1.014) | 1.185  (0.676-2.079) | 1.236  (0.985-1.550) | 1.204  (0.867-1.167) |
| WHRadjBMI (0.1 unit) | Rate^a^  (95% CI) | 1.168  (1.030-1.326) | 1.008  (0.995-1.021) | 0.857  (0.506-1.453) | 1.138  (0.933-1.389) | 1.084  (0.798-1.475) |
| WHRadjBMI (SD) | Rate^a^   (95% CI) | 1.151  (1.028-1.287) | 1.008  (0.995-1.021) | 0.870  (0.541-1.400) | 1.124  (0.941-1.343) | 1.076  (0.819-1.414) |
| **NOTES TO TABLE**: MR-Egger (random effects), IVW (random effects, exact weights), weighted median and weighted mode analyses of BMI (64 SNPs), WHR (34 SNPs) and WHRadjBMI (45 SNPs) on hospital admission rate per year in UK Biobank participants of White British ancestry. SNPs with an LD R^2^ < 0.001 have been retained. Rates are given per exposure unit and exposure SD (SD_BMI_=4.6, SD_WHR_=0.07) and 95% confidence intervals (95% CI) are provided.  BMI = body mass index, CI = confidence interval, IVW = inverse variance weighted, MR = Mendelian randomization, SD = standard deviation, WHR = waist-hip-ratio, WHRadjBMI = waist-hip-ratio adjusted for BMI  a) Adjusted for sex, age and the first 40 genetic principle components. Estimates (with corresponding 95% CIs) represent the fold increase in yearly hospital admission rate per BMI SD (4.6 kg/m^2^) and per WHR SD (0.07). Estimates per BMI and WHR unit are given in Supplementary Table … | | | | | | |

| **Table S9.** MR-Egger (random effects), IVW (random effects, exact weights), weighted median and weighted mode analyses of BMI (64 SNPs), WHR (34 SNPs) and WHRadjBMI (45 SNPs) on hospital admission rate per year in UK Biobank participants of White British ancestry. SNPs with an LD R^2^ < 0.001 have been retained. Rates are given per exposure unit and exposure SD (SD_BMI_=4.6, SD_WHR_=0.07) and 95% confidence intervals (95% CI) are provided. Outliers identified in Figures S1, S2 and S3 were excluded as a sensitivity analysis (3, 4 and 4 SNPs for BMI, WHR and WHRadjBMI, respectively). | | | | | | |
| --- | --- | --- | --- | --- | --- | --- |
|  |  |  | MR-Egger | |  |  |
|  |  | IVW (random effects, exact weights) | Intercept | Slope | Penalized weighted median | Weighted mode |
| BMI (unit) | Rate^a^  (95% CI) | 1.018  (1.002-1.035) | 1.004  (0.997-1.011) | 0.992  (0.941-1.046) | 1.019  (0.983-1.058) | 1.020  (0.978-1.063) |
| BMI (SD) | Rate^a^  (95% CI) | 1.089  (1.011-1.174) | 1.004  (0.997-1.011) | 0.963  (0.750-1.236) | 1.098  (0.931-1.294) | 1.098  (0.905-1.329) |
| WHR (0.1 unit) | Rate^a^   (95% CI) | 1.166  (1.031-1.320) | 1.000  (0.986-1.017) | 1.115  (0.550-2.258) | 1.251  (0.978-1.600) | 1.234  (0.855-1.782) |
| WHR (SD) | Rate^a^   (95% CI) | 1.149  (1.029-1.283) | 1.000  (0.986-1.017) | 1.103  (0.584-2.084) | 1.224  (0.975-1.536) | 1.209  (0.861-1.698) |
| WHRadjBMI (0.1 unit) | Rate^a^  (95% CI) | 1.137  (1.024-1.264) | 1.002  (0.988-1.015) | 1.070  (0.624-1.834) | 1.139  (0.938-1.384) | 1.089  (0.792-1.497) |
| WHRadjBMI (SD) | Rate^a^   (95% CI) | 1.123  (1.015-1.243) | 1.002  (0.988-1.015) | 1.063  (0.654-1.728) | 1.125  (0.945-1.338) | 1.080  (0.792-1.473) |
| BMI = body mass index, CI = confidence interval, IVW = inverse variance weighted, LD = linkage disequilibrium, MR = Mendelian randomization, SD = standard deviation, WHR = waist-hip-ratio  a) Adjusted for sex, age and the first 40 genetic principle components. Estimates (with corresponding 95% CIs) represent the fold increase in yearly hospital admission rate per BMI unit (1 kg/m^2^) and SD (4.6 kg/m^2^) and per 0.1 WHR unit and SD (0.07) | | | | | | |

| **Table S10.** Multivariable two-sample MR IVW estimates for the effect of BMI and WHR (70 SNPs) on yearly hospital admission rate in UK Biobank participants of White British ancestry. Rates and 95% confidence intervals (95% CI) are given. Effect estimates are provided per BMI SD (SD_BMI_=4.6) and per WHR SD (SD_WHR_=0.07). | | |
| --- | --- | --- |
|  | **Rate** | **95% CI** |
| BMI (unit) | 0.997 | (0.966-1.029) |
| BMI (SD) | 0.986 | (0.850-1.143) |
| WHR (0.1 unit) | 1.335 | (1.024-1.739) |
| WHR (SD) | 1.297 | (1.022-1.647) |
| **NOTES TO** TABLE: SNPs with an LD R^2^ < 0.001 were retained. Rates are given per exposure unit and exposure SD (SD_BMI_=4.6, SD_WHR_=0.07) and 95% confidence intervals (95% CI) are provided.  BMI = body mass index, CI = confidence interval, IVW = inverse variance weighted, LD = linkage disequilibrium, MR = Mendelian randomization, SD = standard deviation, WHR = waist-hip-ratio  a) Adjusted for sex, age and the first 40 genetic principal components. Estimates (with corresponding 95% CIs) represent the fold increase in yearly hospital admission rate per BMI unit (1 kg/m^2^) and SD (4.6 kg/m^2^) and per 0.1 WHR unit and SD (0.07) | | |

| **Table S11.** BMI-SNP and hospital admission count-SNP associations as used for the univariate two-sample MR analysis of the effect of BMI on yearly hospital admission rate. The BMI-SNP association coefficients were used as external weights for the genetic risk score in the one-sample MR analysis. | | | | | | | | | | | |
| --- | --- | --- | --- | --- | --- | --- | --- | --- | --- | --- | --- |
|  |  |  |  | **BMI-SNP associations (European ancestry GIANT consortium meta-analysis, Locke *et al.* (2016)** | | | | **Hospital admission count – SNP associations (UK Biobank)** | | | |
| **SNP** | **EA^b^** | **OA** | **EAF** | **β^c^** | **SE** | ***N*** | ***P*** | **β^d^** | **SE** | ***N*** | ***P*** |
| rs1000940 | G | A | 0.320 | 0.019 | 0.003 | 321836 | 1.28E-08 | -0.012 | 0.011 | 310537 | 9.25E-09 |
| rs10132280 | C | A | 0.682 | 0.023 | 0.003 | 321797 | 1.14E-11 | 0.003 | 0.011 | 310537 | 1.76E-01 |
| rs1016287 | T | C | 0.287 | 0.023 | 0.003 | 321969 | 2.25E-11 | 0.015 | 0.011 | 310537 | 9.98E-14 |
| rs10182181 | G | A | 0.462 | 0.031 | 0.003 | 321759 | 8.78E-24 | -0.009 | 0.010 | 310537 | 1.11E-06 |
| rs10733682 | A | G | 0.478 | 0.017 | 0.003 | 320727 | 1.83E-08 | -0.003 | 0.010 | 310537 | 1.24E-01 |
| rs10938397 | G | A | 0.434 | 0.040 | 0.003 | 320955 | 3.21E-38 | -0.004 | 0.010 | 310537 | 5.44E-02 |
| rs10968576 | G | A | 0.320 | 0.025 | 0.003 | 322061 | 6.61E-14 | -0.001 | 0.011 | 310537 | 6.46E-01 |
| rs11030104 | A | G | 0.792 | 0.041 | 0.004 | 322103 | 5.56E-28 | 0.014 | 0.013 | 310537 | 1.43E-09 |
| rs11057405 | G | A | 0.901 | 0.031 | 0.006 | 314111 | 2.02E-08 | 0.009 | 0.017 | 310537 | 3.95E-03 |
| rs11126666^a^ | A | G | 0.283 | 0.021 | 0.003 | 321979 | 1.33E-09 | -0.007 | 0.012 | 310537 | 1.11E-03 |
| rs11165643 | T | C | 0.583 | 0.022 | 0.003 | 320730 | 2.07E-12 | 0.012 | 0.011 | 310537 | 5.15E-10 |
| rs11191560^a^ | C | T | 0.089 | 0.031 | 0.005 | 321893 | 8.45E-09 | 0.020 | 0.019 | 310537 | 1.45E-08 |
| rs11583200^a^ | C | T | 0.396 | 0.018 | 0.003 | 322095 | 1.48E-08 | -0.012 | 0.011 | 310537 | 1.10E-10 |
| rs1167827 | G | A | 0.553 | 0.020 | 0.003 | 306238 | 6.33E-10 | -0.004 | 0.010 | 310537 | 4.97E-02 |
| rs11688816^a^ | G | A | 0.525 | 0.017 | 0.003 | 322051 | 1.89E-08 | 0.000 | 0.010 | 310537 | 9.02E-01 |
| rs11727676 | T | C | 0.910 | 0.036 | 0.006 | 296401 | 2.55E-08 | -0.011 | 0.017 | 310537 | 8.04E-04 |
| rs11847697^a^ | T | C | 0.042 | 0.049 | 0.008 | 306243 | 3.99E-09 | -0.014 | 0.025 | 310537 | 1.68E-03 |
| rs12286929 | G | A | 0.523 | 0.022 | 0.003 | 321903 | 1.31E-12 | 0.016 | 0.010 | 310537 | 1.37E-18 |
| rs12401738 | A | G | 0.352 | 0.021 | 0.003 | 322070 | 1.15E-10 | 0.004 | 0.011 | 310537 | 3.01E-02 |
| rs12429545 | A | G | 0.133 | 0.033 | 0.005 | 312934 | 1.09E-12 | -0.003 | 0.016 | 310537 | 2.15E-01 |
| rs12446632 | G | A | 0.865 | 0.040 | 0.005 | 316758 | 1.48E-18 | -0.005 | 0.015 | 310537 | 3.93E-02 |
| rs12566985^a^ | G | A | 0.446 | 0.024 | 0.003 | 319282 | 3.28E-15 | 0.006 | 0.010 | 310537 | 6.04E-04 |
| rs12885454^a^ | C | A | 0.642 | 0.021 | 0.003 | 320823 | 1.94E-10 | -0.004 | 0.011 | 310537 | 2.07E-02 |
| rs12940622 | G | A | 0.575 | 0.018 | 0.003 | 322032 | 2.49E-09 | -0.022 | 0.010 | 310537 | 8.00E-33 |
| rs13021737 | G | A | 0.828 | 0.060 | 0.004 | 318287 | 1.11E-50 | 0.005 | 0.014 | 310537 | 4.17E-02 |
| rs13078960 | G | T | 0.196 | 0.030 | 0.004 | 322135 | 1.74E-14 | -0.011 | 0.013 | 310537 | 2.48E-06 |
| rs13107325 | T | C | 0.072 | 0.048 | 0.007 | 321461 | 1.83E-12 | -0.011 | 0.020 | 310537 | 2.57E-03 |
| rs13191362 | A | G | 0.879 | 0.028 | 0.005 | 321902 | 7.34E-09 | -0.002 | 0.016 | 310537 | 4.12E-01 |
| rs1516725 | C | T | 0.872 | 0.045 | 0.005 | 320644 | 1.89E-22 | 0.005 | 0.015 | 310537 | 5.97E-02 |
| rs1528435 | T | C | 0.631 | 0.018 | 0.003 | 321924 | 1.20E-08 | -0.009 | 0.011 | 310537 | 9.40E-07 |
| rs1558902 | A | T | 0.415 | 0.082 | 0.003 | 320073 | 7.51E-153 | 0.008 | 0.011 | 310537 | 2.53E-05 |
| rs16851483 | T | G | 0.066 | 0.048 | 0.008 | 233929 | 3.55E-10 | -0.008 | 0.021 | 310537 | 2.41E-02 |
| rs16951275 | T | C | 0.784 | 0.031 | 0.004 | 322098 | 1.91E-17 | 0.007 | 0.012 | 310537 | 6.99E-04 |
| rs17001654 | G | C | 0.153 | 0.031 | 0.005 | 233722 | 7.76E-09 | 0.015 | 0.015 | 310537 | 1.65E-08 |
| rs17024393 | C | T | 0.040 | 0.066 | 0.009 | 297874 | 7.03E-14 | 0.016 | 0.032 | 310537 | 4.78E-03 |
| rs17094222 | C | T | 0.211 | 0.025 | 0.004 | 321770 | 5.94E-11 | 0.001 | 0.013 | 310537 | 7.17E-01 |
| rs17405819 | T | C | 0.700 | 0.022 | 0.003 | 322085 | 2.07E-11 | 0.002 | 0.011 | 310537 | 4.39E-01 |
| rs17724992 | A | G | 0.746 | 0.019 | 0.004 | 319588 | 3.42E-08 | 0.020 | 0.012 | 310537 | 3.19E-22 |
| rs1808579 | C | T | 0.534 | 0.017 | 0.003 | 322032 | 4.17E-08 | 0.001 | 0.010 | 310537 | 4.37E-01 |
| rs1928295 | T | C | 0.548 | 0.019 | 0.003 | 321979 | 7.91E-10 | 0.001 | 0.010 | 310537 | 5.46E-01 |
| rs2033529 | G | A | 0.293 | 0.019 | 0.003 | 321917 | 1.39E-08 | -0.002 | 0.011 | 310537 | 3.73E-01 |
| rs2033732 | C | T | 0.747 | 0.019 | 0.004 | 321406 | 4.89E-08 | -0.000 | 0.012 | 310537 | 9.30E-01 |
| rs205262 | G | A | 0.273 | 0.022 | 0.004 | 315542 | 1.75E-10 | 0.025 | 0.012 | 310537 | 7.63E-32 |
| rs2075650 | A | G | 0.848 | 0.026 | 0.005 | 308408 | 1.25E-08 | -0.009 | 0.015 | 310537 | 4.83E-04 |
| rs2112347 | T | G | 0.629 | 0.026 | 0.003 | 322019 | 6.19E-17 | 0.013 | 0.011 | 310537 | 2.56E-11 |
| rs2121279 | T | C | 0.152 | 0.025 | 0.004 | 322065 | 2.31E-08 | 0.015 | 0.015 | 310537 | 1.86E-08 |
| rs2176598 | T | C | 0.251 | 0.020 | 0.004 | 316848 | 2.97E-08 | 0.004 | 0.012 | 310537 | 8.57E-02 |
| rs2207139 | G | A | 0.177 | 0.045 | 0.004 | 322019 | 4.13E-29 | 0.009 | 0.014 | 310537 | 1.68E-04 |
| rs2245368 | C | T | 0.180 | 0.032 | 0.006 | 205675 | 3.19E-08 | 0.008 | 0.014 | 310537 | 1.00E-03 |
| rs2287019^a^ | C | T | 0.804 | 0.036 | 0.004 | 300921 | 4.59E-18 | 0.013 | 0.014 | 310537 | 8.26E-08 |
| rs2365389 | C | T | 0.582 | 0.020 | 0.003 | 316768 | 1.63E-10 | -0.007 | 0.011 | 310537 | 3.20E-04 |
| rs2650492 | A | G | 0.303 | 0.021 | 0.004 | 319464 | 1.92E-09 | 0.004 | 0.011 | 310537 | 6.54E-02 |
| rs2820292 | C | A | 0.555 | 0.020 | 0.003 | 321707 | 1.83E-10 | 0.003 | 0.010 | 310537 | 6.24E-02 |
| rs29941 | G | A | 0.669 | 0.018 | 0.003 | 321970 | 2.41E-08 | -0.001 | 0.011 | 310537 | 5.97E-01 |
| rs3101336 | C | T | 0.613 | 0.033 | 0.003 | 316872 | 2.66E-26 | 0.005 | 0.011 | 310537 | 4.49E-03 |
| rs3736485 | A | G | 0.454 | 0.018 | 0.003 | 321398 | 7.41E-09 | 0.017 | 0.010 | 310537 | 1.62E-19 |
| rs3810291^a^ | A | G | 0.666 | 0.028 | 0.004 | 296261 | 4.81E-15 | 0.011 | 0.011 | 310537 | 1.46E-08 |
| rs3817334 | T | C | 0.407 | 0.026 | 0.003 | 321959 | 5.15E-17 | -0.004 | 0.010 | 310537 | 5.00E-02 |
| rs3849570 | A | C | 0.359 | 0.019 | 0.003 | 284339 | 2.60E-08 | 0.010 | 0.011 | 310537 | 2.95E-07 |
| rs3888190^a^ | A | C | 0.403 | 0.031 | 0.003 | 321930 | 3.14E-23 | 0.013 | 0.011 | 310537 | 1.71E-11 |
| rs4256980 | G | C | 0.646 | 0.021 | 0.003 | 320028 | 2.90E-11 | 0.003 | 0.011 | 310537 | 8.63E-02 |
| rs4740619 | T | C | 0.542 | 0.018 | 0.003 | 321887 | 4.56E-09 | 0.023 | 0.010 | 310537 | 3.67E-36 |
| rs543874 | G | A | 0.193 | 0.048 | 0.004 | 322008 | 2.62E-35 | -0.005 | 0.013 | 310537 | 3.75E-02 |
| rs6477694 | C | T | 0.365 | 0.017 | 0.003 | 322048 | 2.67E-08 | 0.013 | 0.011 | 310537 | 5.67E-12 |
| rs6567160^a^ | C | T | 0.236 | 0.056 | 0.004 | 321958 | 3.93E-53 | 0.012 | 0.012 | 310537 | 9.65E-09 |
| rs657452 | A | G | 0.394 | 0.023 | 0.003 | 313651 | 5.48E-13 | 0.014 | 0.011 | 310537 | 9.58E-14 |
| rs6804842 | G | A | 0.575 | 0.019 | 0.003 | 321463 | 2.48E-09 | -0.015 | 0.010 | 310537 | 2.87E-16 |
| rs7138803 | A | G | 0.384 | 0.032 | 0.003 | 322092 | 8.15E-24 | -0.016 | 0.011 | 310537 | 4.60E-16 |
| rs7141420 | T | C | 0.527 | 0.024 | 0.003 | 321970 | 1.23E-14 | 0.004 | 0.010 | 310537 | 2.70E-02 |
| rs7243357 | T | G | 0.812 | 0.022 | 0.004 | 322107 | 3.86E-08 | 0.028 | 0.014 | 310537 | 6.55E-31 |
| rs758747 | T | C | 0.265 | 0.023 | 0.004 | 308688 | 7.47E-10 | -0.009 | 0.012 | 310537 | 5.07E-06 |
| rs7599312 | G | A | 0.724 | 0.022 | 0.003 | 322024 | 1.17E-10 | 0.006 | 0.012 | 310537 | 3.37E-03 |
| rs7899106 | G | A | 0.052 | 0.040 | 0.007 | 321770 | 2.96E-08 | -0.009 | 0.024 | 310537 | 3.19E-02 |
| rs7903146 | C | T | 0.713 | 0.023 | 0.003 | 322130 | 1.11E-11 | -0.000 | 0.011 | 310537 | 9.53E-01 |
| rs9400239 | C | T | 0.688 | 0.019 | 0.003 | 321988 | 1.61E-08 | 0.017 | 0.011 | 310537 | 7.57E-18 |
| rs9925964^a^ | A | G | 0.620 | 0.019 | 0.003 | 318385 | 8.11E-10 | 0.011 | 0.011 | 310537 | 9.47E-09 |
| BMI = body mass index, EA = effect allele, EAF = effect allele frequency, LD = linkage disequilibrium, MR = Mendelian randomization, OA = other allele, SE = standard error  a) These 12 SNPs exceeded the LD threshold (R^2^<0.001) and were excluded from the two-sample summary MR analysis  b) The effect allele (EA) is the BMI increasing allele  c) Coefficients are given per BMI SD (4.6 kg/m^2^)  d) Coefficients are given on the logarithmic scale, obtained from a quasi-Poisson regression of hospital admission count on the relevant SNP, using person-years on study as an offset and adjusting for age, sex and the first 40 genetic principle components | | | | | | | | | | | |

| **Table S12.** WHR-SNP and hospital admission count-SNP associations as used for the univariate two-sample MR analysis of the effect of WHR on yearly hospital admission rate. The WHR-SNP association coefficients were used as external weights for the genetic risk score in the one-sample MR analysis. | | | | | | | | | | | |
| --- | --- | --- | --- | --- | --- | --- | --- | --- | --- | --- | --- |
|  |  |  |  | **WHR-SNP associations (European ancestry GIANT consortium meta-analysis, Shungin *et al.* (2015)** | | | | **Hospital admission count – SNP associations (UK Biobank)** | | | |
| **SNP** | **EA^b^** | **OA** | **EAF** | **β^c^** | **SE** | ***N*** | ***P*** | **β^d^** | **SE** | ***N*** | ***P*** |
| rs1011731^a^ | G | A | 0.427 | 0.019 | 0.003 | 212094 | 1.07E-08 | -0.010 | 0.010 | 310537 | 7.08E-08 |
| rs10195252 | T | C | 0.590 | 0.020 | 0.003 | 211907 | 2.57E-09 | 0.005 | 0.011 | 310537 | 9.27E-03 |
| rs10245353 | A | C | 0.200 | 0.027 | 0.004 | 212151 | 1.57E-10 | 0.006 | 0.013 | 310537 | 9.64E-03 |
| rs1045241 | C | T | 0.714 | 0.015 | 0.004 | 212012 | 5.76E-05 | -0.008 | 0.012 | 310537 | 8.74E-05 |
| rs10804591 | A | C | 0.793 | 0.021 | 0.004 | 212108 | 2.09E-07 | -0.005 | 0.013 | 310537 | 1.63E-02 |
| rs11048470 | T | G | 0.277 | 0.025 | 0.004 | 212159 | 6.33E-12 | -0.001 | 0.012 | 310537 | 6.45E-01 |
| rs1121980 | A | G | 0.433 | 0.043 | 0.003 | 211970 | 1.33E-38 | 0.011 | 0.010 | 310537 | 5.44E-09 |
| rs11663816 | C | T | 0.265 | 0.025 | 0.004 | 212104 | 2.65E-11 | 0.006 | 0.012 | 310537 | 4.99E-03 |
| rs11989744 | C | T | 0.760 | 0.021 | 0.005 | 144546 | 1.30E-05 | 0.007 | 0.012 | 310537 | 5.43E-04 |
| rs12549058 | G | T | 0.084 | 0.040 | 0.006 | 212028 | 3.17E-10 | -0.004 | 0.022 | 310537 | 3.19E-01 |
| rs1294421 | G | T | 0.620 | 0.026 | 0.003 | 212054 | 6.93E-14 | -0.007 | 0.011 | 310537 | 4.15E-04 |
| rs1358980 | T | C | 0.470 | 0.027 | 0.004 | 211037 | 1.98E-14 | 0.014 | 0.010 | 310537 | 1.48E-13 |
| rs1394461 | C | G | 0.251 | 0.017 | 0.005 | 144349 | 4.68E-04 | 0.005 | 0.013 | 310537 | 1.33E-02 |
| rs1440372 | C | T | 0.710 | 0.021 | 0.004 | 210387 | 7.59E-09 | 0.014 | 0.012 | 310537 | 4.20E-11 |
| rs1443512 | A | C | 0.235 | 0.026 | 0.004 | 212153 | 2.76E-11 | 0.001 | 0.013 | 310537 | 7.11E-01 |
| rs1515108 | C | T | 0.377 | 0.007 | 0.003 | 212094 | 3.13E-02 | -0.020 | 0.011 | 310537 | 8.41E-26 |
| rs1569135 | A | G | 0.529 | 0.024 | 0.003 | 212086 | 1.00E-12 | 0.012 | 0.010 | 310537 | 2.61E-10 |
| rs16996700 | T | C | 0.730 | 0.021 | 0.004 | 212159 | 1.60E-08 | 0.014 | 0.011 | 310537 | 8.92E-12 |
| rs17109256 | A | G | 0.219 | 0.023 | 0.004 | 208422 | 3.02E-08 | 0.005 | 0.013 | 310537 | 2.67E-02 |
| rs17451107 | T | C | 0.613 | 0.023 | 0.004 | 211586 | 3.50E-11 | -0.005 | 0.011 | 310537 | 1.26E-02 |
| rs17819328 | G | T | 0.432 | 0.016 | 0.004 | 211496 | 2.29E-06 | 0.029 | 0.010 | 310537 | 2.35E-55 |
| rs2075650 | A | G | 0.848 | 0.029 | 0.005 | 206613 | 6.43E-09 | -0.009 | 0.015 | 310537 | 4.83E-04 |
| rs2179129 | A | G | 0.590 | 0.021 | 0.003 | 212181 | 1.24E-09 | 0.016 | 0.011 | 310537 | 1.54E-18 |
| rs2287019^a^ | C | T | 0.802 | 0.026 | 0.005 | 199713 | 4.34E-09 | 0.013 | 0.014 | 310537 | 8.26E-08 |
| rs2765539 | T | C | 0.735 | 0.027 | 0.004 | 212176 | 1.08E-12 | 0.001 | 0.012 | 310537 | 6.31E-01 |
| rs319564 | C | T | 0.450 | 0.014 | 0.003 | 212137 | 3.42E-05 | 0.012 | 0.010 | 310537 | 4.00E-10 |
| rs3786897 | G | A | 0.417 | 0.022 | 0.003 | 212009 | 3.95E-11 | -0.002 | 0.010 | 310537 | 2.46E-01 |
| rs4471313 | T | G | 0.720 | 0.020 | 0.004 | 180767 | 6.92E-07 | 0.025 | 0.012 | 310537 | 6.19E-33 |
| rs459193 | A | G | 0.264 | 0.026 | 0.004 | 212101 | 6.02E-12 | -0.008 | 0.013 | 310537 | 3.67E-04 |
| rs4640244^a^ | G | A | 0.396 | 0.021 | 0.004 | 198799 | 3.11E-08 | 0.013 | 0.011 | 310537 | 1.06E-11 |
| rs4646404 | G | A | 0.661 | 0.020 | 0.004 | 201330 | 3.81E-07 | -0.002 | 0.011 | 310537 | 3.19E-01 |
| rs4715208^a^ | G | A | 0.740 | 0.019 | 0.004 | 212191 | 7.91E-07 | -0.004 | 0.012 | 310537 | 7.70E-02 |
| rs4846565 | G | A | 0.672 | 0.023 | 0.004 | 212157 | 4.75E-11 | 0.009 | 0.011 | 310537 | 5.30E-06 |
| rs4929927 | G | A | 0.646 | 0.020 | 0.003 | 212152 | 7.60E-09 | 0.004 | 0.011 | 310537 | 4.70E-02 |
| rs7801581^a^ | T | C | 0.244 | 0.023 | 0.004 | 198342 | 4.95E-08 | -0.022 | 0.012 | 310537 | 7.73E-24 |
| rs863750 | T | C | 0.593 | 0.016 | 0.003 | 212134 | 1.56E-06 | 0.003 | 0.011 | 310537 | 1.33E-01 |
| rs929641 | A | G | 0.587 | 0.020 | 0.003 | 212102 | 4.25E-09 | -0.015 | 0.011 | 310537 | 3.58E-16 |
| rs9491696 | G | C | 0.482 | 0.038 | 0.003 | 211988 | 4.88E-30 | 0.005 | 0.010 | 310537 | 1.37E-02 |
| rs9860730 | A | G | 0.703 | 0.023 | 0.004 | 212062 | 2.84E-10 | 0.005 | 0.011 | 310537 | 1.14E-02 |
| EA = effect allele, EAF = effect allele frequency, LD = linkage disequilibrium, MR = Mendelian randomization, OA = other allele, SE = standard error, WHR = waist-hip-ratio  a) These 5 SNPs exceeded the LD threshold (R^2^<0.001) and were excluded from the two-sample summary MR analysis  b) The effect allele (EA) is the WHR increasing allele  c) Coefficients are given per WHR SD (0.07)  d) Coefficients are given on the logarithmic scale, obtained from a quasi-Poisson regression of hospital admission count on the relevant SNP, using person-years on study as an offset and adjusting for age, sex and the first 40 genetic principle components | | | | | | | | | | | |

| **Table S13.** WHRadjBMI-SNP and hospital admission count-SNP associations as used for the univariate two-sample MR analysis of the effect of WHRadjBMI on yearly hospital admission rate. The WHRadjBMI-SNP association coefficients were used as external weights for the genetic risk score in the one-sample MR analysis | | | | | | | | | | | |
| --- | --- | --- | --- | --- | --- | --- | --- | --- | --- | --- | --- |
|  |  |  |  | **WHRadjBMI-SNP associations (European ancestry GIANT consortium meta-analysis, Shungin *et al.* (2015)** | | | | **Hospital admission count – SNP associations (UK Biobank)** | | | |
| **SNP** | **EA^b^** | **OA** | **EAF** | **β^c^** | **SE** | ***N*** | ***P*** | **β^d^** | **SE** | ***N*** | ***P*** |
| rs10195252 | T | C | 0.587 | 0.031 | 0.004 | 142102 | 1.03E-15 | 0.005 | 0.011 | 310537 | 9.27E-03 |
| rs10245353 | A | C | 0.196 | 0.037 | 0.005 | 142708 | 6.88E-14 | 0.006 | 0.013 | 310537 | 9.64E-03 |
| rs1045241 | C | T | 0.714 | 0.022 | 0.004 | 142400 | 3.18E-07 | -0.008 | 0.012 | 310537 | 8.74E-05 |
| rs10804591 | A | C | 0.794 | 0.025 | 0.005 | 142653 | 1.93E-07 | -0.005 | 0.013 | 310537 | 1.63E-02 |
| rs10842707 | T | C | 0.227 | 0.036 | 0.005 | 142708 | 4.39E-15 | -0.003 | 0.013 | 310537 | 1.53E-01 |
| rs10919388 | C | A | 0.721 | 0.025 | 0.004 | 142721 | 5.33E-09 | 0.025 | 0.012 | 310537 | 8.67E-33 |
| rs10991437 | A | C | 0.117 | 0.033 | 0.006 | 142661 | 9.74E-08 | -0.003 | 0.016 | 310537 | 2.41E-01 |
| rs11231693 | A | G | 0.061 | 0.048 | 0.009 | 130856 | 8.20E-08 | 0.008 | 0.023 | 310537 | 3.54E-02 |
| rs12454712 | T | C | 0.615 | 0.017 | 0.006 | 102489 | 3.26E-03 | 0.004 | 0.011 | 310537 | 1.84E-02 |
| rs12608504 | A | G | 0.354 | 0.020 | 0.004 | 142678 | 1.20E-06 | 0.010 | 0.011 | 310537 | 6.65E-07 |
| rs12679556 | G | T | 0.245 | 0.024 | 0.005 | 142669 | 1.12E-07 | 0.006 | 0.012 | 310537 | 3.20E-03 |
| rs1294410 | C | T | 0.633 | 0.034 | 0.004 | 142548 | 1.42E-17 | -0.003 | 0.011 | 310537 | 6.55E-02 |
| rs1358980 | T | C | 0.461 | 0.039 | 0.004 | 139579 | 3.01E-20 | 0.014 | 0.010 | 310537 | 1.48E-13 |
| rs1385167 | G | A | 0.142 | 0.032 | 0.006 | 139368 | 9.40E-09 | -0.028 | 0.015 | 310537 | 7.87E-25 |
| rs1440372 | C | T | 0.705 | 0.023 | 0.004 | 141188 | 7.84E-08 | 0.014 | 0.012 | 310537 | 4.20E-11 |
| rs1443512 | A | C | 0.233 | 0.031 | 0.005 | 142694 | 5.38E-12 | 0.001 | 0.013 | 310537 | 7.11E-01 |
| rs1569135 | A | G | 0.528 | 0.020 | 0.004 | 142674 | 3.58E-07 | 0.012 | 0.010 | 310537 | 2.61E-10 |
| rs17451107 | T | C | 0.614 | 0.027 | 0.004 | 140959 | 1.48E-10 | -0.005 | 0.011 | 310537 | 1.26E-02 |
| rs1776897 | G | T | 0.082 | 0.041 | 0.008 | 110603 | 5.50E-07 | 0.011 | 0.018 | 310537 | 1.24E-03 |
| rs17819328 | G | T | 0.431 | 0.022 | 0.004 | 141503 | 5.82E-08 | 0.029 | 0.010 | 310537 | 2.35E-55 |
| rs1936805 | T | C | 0.509 | 0.042 | 0.004 | 142540 | 6.13E-28 | 0.006 | 0.010 | 310537 | 1.69E-03 |
| rs224333 | G | A | 0.634 | 0.022 | 0.004 | 142206 | 2.76E-07 | 0.002 | 0.011 | 310537 | 2.90E-01 |
| rs2276824 | C | G | 0.433 | 0.022 | 0.004 | 141626 | 7.12E-08 | -0.007 | 0.010 | 310537 | 6.07E-05 |
| rs2294239 | A | G | 0.586 | 0.028 | 0.004 | 142140 | 1.86E-12 | 0.016 | 0.010 | 310537 | 1.26E-17 |
| rs2371767 | G | C | 0.723 | 0.034 | 0.005 | 130378 | 9.49E-14 | 0.000 | 0.012 | 310537 | 8.52E-01 |
| rs2645294 | T | C | 0.575 | 0.034 | 0.004 | 142526 | 4.42E-18 | 0.002 | 0.010 | 310537 | 3.07E-01 |
| rs2820443 | T | C | 0.714 | 0.041 | 0.004 | 142663 | 1.50E-21 | 0.002 | 0.011 | 310537 | 2.86E-01 |
| rs2925979 | T | C | 0.305 | 0.018 | 0.004 | 140533 | 1.44E-05 | 0.010 | 0.011 | 310537 | 2.29E-07 |
| rs303084 | A | G | 0.798 | 0.024 | 0.005 | 142629 | 5.30E-07 | -0.006 | 0.013 | 310537 | 5.39E-03 |
| rs3805389 | A | G | 0.279 | 0.018 | 0.004 | 141909 | 4.16E-05 | 0.011 | 0.012 | 310537 | 3.00E-08 |
| rs4081724 | G | A | 0.856 | 0.031 | 0.006 | 140159 | 1.25E-07 | 0.018 | 0.015 | 310537 | 1.40E-11 |
| rs4646404 | G | A | 0.665 | 0.026 | 0.005 | 131007 | 3.46E-08 | -0.002 | 0.011 | 310537 | 3.19E-01 |
| rs4765219 | C | A | 0.667 | 0.030 | 0.004 | 142549 | 5.99E-14 | 0.005 | 0.011 | 310537 | 1.02E-02 |
| rs6090583 | A | G | 0.474 | 0.020 | 0.004 | 142152 | 2.47E-07 | 0.028 | 0.010 | 310537 | 1.56E-50 |
| rs6556301^a^ | T | G | 0.356 | 0.022 | 0.004 | 140524 | 2.60E-07 | -0.006 | 0.011 | 310537 | 3.89E-03 |
| rs714515^a^ | G | A | 0.428 | 0.031 | 0.004 | 142435 | 6.72E-16 | -0.010 | 0.010 | 310537 | 2.89E-08 |
| rs7705502 | A | G | 0.320 | 0.023 | 0.004 | 142668 | 2.38E-08 | -0.021 | 0.011 | 310537 | 9.98E-26 |
| rs7759742 | A | T | 0.505 | 0.024 | 0.004 | 140972 | 1.50E-09 | 0.013 | 0.010 | 310537 | 6.67E-13 |
| rs7801581^a^ | T | C | 0.242 | 0.027 | 0.005 | 129075 | 5.02E-08 | -0.022 | 0.012 | 310537 | 7.73E-24 |
| rs7830933 | A | G | 0.766 | 0.021 | 0.005 | 142458 | 4.73E-06 | 0.013 | 0.012 | 310537 | 7.00E-10 |
| rs7917772 | A | G | 0.620 | 0.017 | 0.004 | 142345 | 3.20E-05 | 0.003 | 0.011 | 310537 | 9.90E-02 |
| rs8030605 | A | G | 0.150 | 0.029 | 0.006 | 141175 | 1.66E-06 | 0.003 | 0.016 | 310537 | 2.47E-01 |
| rs8042543 | C | T | 0.795 | 0.024 | 0.005 | 141009 | 3.45E-06 | -0.003 | 0.013 | 310537 | 1.36E-01 |
| rs8066985 | A | G | 0.507 | 0.019 | 0.004 | 142684 | 1.28E-06 | 0.001 | 0.010 | 310537 | 8.06E-01 |
| rs905938 | T | C | 0.738 | 0.030 | 0.005 | 140583 | 1.73E-10 | -0.013 | 0.012 | 310537 | 8.45E-10 |
| rs9687846 | A | G | 0.186 | 0.027 | 0.005 | 142400 | 8.28E-08 | -0.002 | 0.013 | 310537 | 4.01E-01 |
| rs979012 | T | C | 0.347 | 0.026 | 0.004 | 142646 | 4.22E-10 | 0.013 | 0.011 | 310537 | 5.13E-12 |
| rs9991328 | T | C | 0.480 | 0.019 | 0.004 | 142624 | 5.87E-07 | -0.014 | 0.010 | 310537 | 4.62E-14 |
| BMI = body mass index, EA = effect allele, EAF = effect allele frequency, LD = linkage disequilibrium, MR = Mendelian randomization, OA = other allele, SE = standard error, WHRadjBMI = waist-hip-ratio adjusted for BMI  a) These 3 SNPs exceeded the LD threshold (R^2^<0.001) and were excluded from the two-sample summary MR analysis  b) The effect allele (EA) is the WHR increasing allele  c) Coefficients are given per WHR SD (0.07)  d) Coefficients are given on the logarithmic scale, obtained from a quasi-Poisson regression of hospital admission count on the relevant SNP, using person-years on study as an offset and adjusting for age, sex and the first 40 PCAs | | | | | | | | | | | |

| **Table S14.** BMI-SNP, WHR-SNP and hospital admission count-SNP associations as used for the multivariable two-sample MR analysis of the effect of BMI and WHR on yearly hospital admission rate. Given are the 70 SNPs remaining after LD correction (R^2^<0.001) to the joint set of BMI and WHR SNPs. | | | | | | | | | | | | | | |
| --- | --- | --- | --- | --- | --- | --- | --- | --- | --- | --- | --- | --- | --- | --- |
|  |  |  |  | **BMI-SNP associations (European ancestry GIANT consortium meta-analysis, Locke *et al.* (2016)** | | | | **WHR-SNP associations (European ancestry GIANT consortium meta-analysis, Shungin *et al.* (2015)** | | | | **Hospital admission count – SNP associations (UK Biobank, *N*=310537)** | | |
| **SNP** | **EA** | **OA** | **EAF** | **β^a^** | **SE** | ***P*** | ***N*** | **β^a^** | **SE** | ***P*** | ***N*** | **β^b^** | **SE** | ***P*** |
| rs1000940 | G | A | 0.225 | 0.019 | 0.003 | 1.28E-08 | 321836 | 0.008 | 0.004 | 2.30E-02 | 211915 | -0.012 | 0.011 | 9.25E-09 |
| rs1011731 | G | A | 0.458 | -0.006 | 0.003 | 3.88E-02 | 321942 | 0.019 | 0.003 | 1.10E-08 | 212094 | -0.010 | 0.010 | 7.08E-08 |
| rs10132280 | A | C | 0.333 | -0.023 | 0.003 | 1.14E-11 | 321797 | -0.012 | 0.004 | 8.40E-04 | 212105 | 0.003 | 0.011 | 1.76E-01 |
| rs1016287 | T | C | 0.325 | 0.023 | 0.003 | 2.25E-11 | 321969 | 0.013 | 0.004 | 6.20E-04 | 212119 | 0.015 | 0.011 | 9.98E-14 |
| rs10182181 | A | G | 0.500 | -0.031 | 0.003 | 8.78E-24 | 321759 | -0.005 | 0.003 | 1.60E-01 | 211882 | -0.009 | 0.010 | 1.11E-06 |
| rs10245353 | A | C | 0.183 | -0.002 | 0.004 | 5.88E-01 | 322088 | 0.027 | 0.004 | 1.60E-10 | 212151 | 0.006 | 0.013 | 9.64E-03 |
| rs10733682 | A | G | 0.425 | 0.017 | 0.003 | 1.83E-08 | 320727 | 0.010 | 0.003 | 2.90E-03 | 211864 | -0.003 | 0.010 | 1.24E-01 |
| rs10938397 | A | G | 0.567 | -0.040 | 0.003 | 3.20E-38 | 320955 | -0.018 | 0.003 | 2.40E-07 | 211843 | -0.004 | 0.010 | 5.44E-02 |
| rs10968576 | G | A | 0.292 | 0.025 | 0.003 | 6.61E-14 | 322061 | 0.017 | 0.004 | 2.40E-06 | 212162 | -0.001 | 0.011 | 6.46E-01 |
| rs11030104 | A | G | 0.800 | 0.041 | 0.004 | 5.56E-28 | 322103 | 0.020 | 0.004 | 8.50E-07 | 212160 | 0.014 | 0.013 | 1.43E-09 |
| rs11048470 | T | G | 0.233 | -0.009 | 0.003 | 6.35E-03 | 322043 | 0.025 | 0.004 | 6.30E-12 | 212159 | -0.001 | 0.012 | 6.45E-01 |
| rs11165643 | C | T | 0.425 | -0.022 | 0.003 | 2.07E-12 | 320730 | -0.009 | 0.003 | 6.60E-03 | 212148 | 0.012 | 0.011 | 5.15E-10 |
| rs1167827 | A | G | 0.458 | -0.020 | 0.003 | 6.33E-10 | 306238 | -0.006 | 0.004 | 8.00E-02 | 204123 | -0.004 | 0.010 | 4.97E-02 |
| rs11727676 | C | T | 0.075 | 0.036 | 0.006 | 2.55E-08 | 296401 | 0.005 | 0.007 | 4.30E-01 | 191737 | -0.011 | 0.017 | 8.04E-04 |
| rs12286929 | G | A | 0.433 | 0.022 | 0.003 | 1.31E-12 | 321903 | 0.010 | 0.003 | 4.10E-03 | 212086 | 0.016 | 0.010 | 1.37E-18 |
| rs12429545 | G | A | 0.900 | 0.033 | 0.005 | 1.09E-12 | 312934 | -0.015 | 0.005 | 2.90E-03 | 203113 | -0.003 | 0.016 | 2.15E-01 |
| rs12940622 | A | G | 0.458 | -0.018 | 0.003 | 2.49E-09 | 322032 | -0.007 | 0.003 | 2.60E-02 | 212119 | -0.007 | 0.011 | 8.00E-33 |
| rs1294421 | G | T | 0.600 | -0.005 | 0.003 | 1.42E-01 | 321751 | 0.025 | 0.003 | 6.90E-14 | 212054 | 0.005 | 0.014 | 4.15E-04 |
| rs13021737 | A | G | 0.125 | -0.060 | 0.004 | 1.11E-50 | 318287 | -0.023 | 0.004 | 1.90E-07 | 209902 | -0.011 | 0.013 | 4.17E-02 |
| rs13078960 | T | G | 0.817 | -0.030 | 0.004 | 1.74E-14 | 322135 | -0.010 | 0.004 | 2.10E-02 | 212190 | -0.011 | 0.020 | 2.48E-06 |
| rs13107325 | C | T | 0.883 | -0.048 | 0.007 | 1.82E-12 | 321461 | 0.000 | 0.007 | 1.00E+00 | 211675 | -0.002 | 0.016 | 2.57E-03 |
| rs13191362 | A | G | 0.800 | 0.028 | 0.005 | 7.34E-09 | 321902 | 0.018 | 0.005 | 6.00E-04 | 212035 | 0.005 | 0.015 | 4.12E-01 |
| rs1516725 | T | C | 0.092 | -0.045 | 0.005 | 1.89E-22 | 320644 | -0.014 | 0.005 | 4.40E-03 | 210725 | 0.012 | 0.010 | 5.97E-02 |
| rs1569135 | A | G | 0.533 | 0.011 | 0.003 | 5.24E-04 | 322000 | 0.024 | 0.003 | 1.00E-12 | 212086 | -0.008 | 0.021 | 2.61E-10 |
| rs16851483 | G | T | 0.908 | -0.048 | 0.008 | 3.55E-10 | 233929 | -0.019 | 0.008 | 2.20E-02 | 144591 | 0.007 | 0.012 | 2.41E-02 |
| rs16951275 | C | T | 0.225 | -0.031 | 0.004 | 1.91E-17 | 322098 | -0.016 | 0.004 | 5.00E-05 | 212172 | 0.014 | 0.011 | 6.99E-04 |
| rs16996700 | T | C | 0.700 | 0.018 | 0.003 | 1.74E-07 | 322063 | 0.021 | 0.004 | 1.60E-08 | 212159 | 0.015 | 0.015 | 8.92E-12 |
| rs17001654 | C | G | 0.842 | -0.031 | 0.005 | 7.76E-09 | 233722 | -0.010 | 0.006 | 9.30E-02 | 144378 | 0.016 | 0.032 | 1.65E-08 |
| rs17024393 | C | T | 0.042 | 0.066 | 0.009 | 7.03E-14 | 297874 | 0.029 | 0.009 | 1.80E-03 | 189151 | 0.001 | 0.013 | 4.78E-03 |
| rs17094222 | C | T | 0.208 | 0.025 | 0.004 | 5.94E-11 | 321770 | 0.013 | 0.004 | 2.00E-03 | 212078 | 0.002 | 0.011 | 7.17E-01 |
| rs17405819 | C | T | 0.367 | -0.022 | 0.003 | 2.07E-11 | 322085 | -0.002 | 0.004 | 6.80E-01 | 212166 | -0.005 | 0.011 | 4.39E-01 |
| rs17451107 | T | C | 0.625 | 0.005 | 0.003 | 1.58E-01 | 320725 | 0.023 | 0.004 | 3.50E-11 | 211586 | 0.020 | 0.012 | 1.26E-02 |
| rs17724992 | A | G | 0.692 | 0.019 | 0.004 | 3.42E-08 | 319588 | 0.019 | 0.004 | 9.30E-07 | 210660 | 0.001 | 0.010 | 3.19E-22 |
| rs1808579 | T | C | 0.475 | -0.017 | 0.003 | 4.17E-08 | 322032 | -0.004 | 0.003 | 1.80E-01 | 212105 | 0.001 | 0.010 | 4.37E-01 |
| rs1928295 | C | T | 0.425 | -0.019 | 0.003 | 7.91E-10 | 321979 | -0.012 | 0.003 | 3.90E-04 | 212089 | -0.002 | 0.011 | 5.46E-01 |
| rs2033529 | G | A | 0.258 | 0.019 | 0.003 | 1.39E-08 | 321917 | 0.016 | 0.004 | 6.00E-06 | 212146 | -0.000 | 0.012 | 3.73E-01 |
| rs2033732 | C | T | 0.758 | 0.019 | 0.004 | 4.89E-08 | 321406 | 0.009 | 0.004 | 1.80E-02 | 211985 | 0.025 | 0.012 | 9.30E-01 |
| rs205262 | A | G | 0.733 | -0.022 | 0.004 | 1.75E-10 | 315542 | 0.000 | 0.004 | 9.80E-01 | 205608 | 0.013 | 0.011 | 7.63E-32 |
| rs2112347 | G | T | 0.375 | -0.026 | 0.003 | 6.19E-17 | 322019 | -0.013 | 0.003 | 2.50E-04 | 212132 | 0.015 | 0.015 | 2.56E-11 |
| rs2121279 | T | C | 0.117 | 0.024 | 0.004 | 2.31E-08 | 322065 | 0.010 | 0.005 | 4.20E-02 | 212154 | 0.004 | 0.012 | 1.86E-08 |
| rs2176598 | T | C | 0.200 | 0.020 | 0.004 | 2.97E-08 | 316848 | 0.016 | 0.004 | 3.50E-05 | 206987 | 0.016 | 0.011 | 8.57E-02 |
| rs2179129 | A | G | 0.550 | -0.002 | 0.003 | 5.87E-01 | 321616 | 0.021 | 0.003 | 1.20E-09 | 212181 | 0.009 | 0.014 | 1.54E-18 |
| rs2207139 | G | A | 0.100 | 0.045 | 0.004 | 4.13E-29 | 322019 | 0.025 | 0.004 | 1.40E-08 | 212163 | 0.008 | 0.014 | 1.68E-04 |
| rs2245368 | T | C | 0.758 | -0.032 | 0.006 | 3.19E-08 | 205675 | -0.009 | 0.006 | 1.30E-01 | 133817 | 0.013 | 0.014 | 1.00E-03 |
| rs2287019 | C | T | 0.850 | 0.036 | 0.004 | 4.58E-18 | 300921 | 0.026 | 0.004 | 4.30E-09 | 199713 | 0.013 | 0.014 | 8.26E-08 |
| rs2365389 | C | T | 0.658 | 0.020 | 0.003 | 1.63E-10 | 316768 | 0.008 | 0.003 | 2.20E-02 | 208487 | -0.007 | 0.011 | 3.20E-04 |
| rs2820292 | A | C | 0.492 | -0.020 | 0.003 | 1.83E-10 | 321707 | -0.011 | 0.003 | 7.80E-04 | 212043 | 0.003 | 0.010 | 6.24E-02 |
| rs3101336 | T | C | 0.351 | -0.033 | 0.003 | 2.66E-26 | 316872 | -0.016 | 0.003 | 3.60E-06 | 206980 | 0.005 | 0.011 | 4.49E-03 |
| rs3736485 | A | G | 0.425 | 0.018 | 0.003 | 7.41E-09 | 321398 | 0.015 | 0.003 | 6.60E-06 | 212010 | 0.017 | 0.010 | 1.62E-19 |
| rs3786897 | G | A | 0.408 | 0.007 | 0.003 | 3.43E-02 | 321528 | 0.022 | 0.003 | 4.00E-11 | 212009 | -0.002 | 0.010 | 2.46E-01 |
| rs3817334 | C | T | 0.550 | -0.026 | 0.003 | 5.14E-17 | 321959 | -0.009 | 0.003 | 6.10E-03 | 212046 | -0.004 | 0.010 | 5.00E-02 |
| rs3849570 | A | C | 0.367 | 0.019 | 0.003 | 2.60E-08 | 284339 | 0.011 | 0.004 | 2.20E-03 | 182853 | 0.010 | 0.011 | 2.95E-07 |
| rs3888190 | A | C | 0.358 | 0.031 | 0.003 | 3.14E-23 | 321930 | 0.016 | 0.003 | 1.40E-06 | 212068 | 0.013 | 0.011 | 1.71E-11 |
| rs4256980 | G | C | 0.725 | 0.021 | 0.003 | 2.90E-11 | 320028 | 0.019 | 0.003 | 5.30E-08 | 210672 | 0.003 | 0.011 | 8.63E-02 |
| rs459193 | A | G | 0.217 | 0.007 | 0.004 | 4.29E-02 | 321858 | 0.026 | 0.004 | 6.00E-12 | 212101 | -0.008 | 0.013 | 3.67E-04 |
| rs4640244 | G | A | 0.375 | 0.016 | 0.003 | 4.57E-06 | 305292 | 0.021 | 0.004 | 3.10E-08 | 198799 | 0.013 | 0.011 | 1.06E-11 |
| rs4740619 | T | C | 0.533 | 0.018 | 0.003 | 4.56E-09 | 321887 | 0.011 | 0.003 | 1.10E-03 | 212076 | 0.023 | 0.010 | 3.67E-36 |
| rs543874 | G | A | 0.267 | 0.048 | 0.004 | 2.62E-35 | 322008 | 0.020 | 0.004 | 2.00E-06 | 212160 | -0.005 | 0.013 | 3.75E-02 |
| rs6477694 | C | T | 0.358 | 0.017 | 0.003 | 2.67E-08 | 322048 | 0.015 | 0.003 | 1.50E-05 | 212114 | 0.013 | 0.011 | 5.67E-12 |
| rs6567160 | C | T | 0.283 | 0.056 | 0.004 | 3.93E-53 | 321958 | 0.025 | 0.004 | 5.90E-10 | 212147 | 0.012 | 0.012 | 9.65E-09 |
| rs657452 | A | G | 0.417 | 0.023 | 0.003 | 5.48E-13 | 313651 | 0.014 | 0.004 | 4.20E-05 | 204624 | 0.014 | 0.011 | 9.58E-14 |
| rs6804842 | A | G | 0.425 | -0.018 | 0.003 | 2.48E-09 | 321463 | -0.006 | 0.003 | 5.80E-02 | 212015 | -0.015 | 0.010 | 2.87E-16 |
| rs7138803 | G | A | 0.558 | -0.032 | 0.003 | 8.15E-24 | 322092 | -0.014 | 0.004 | 3.50E-05 | 212167 | -0.016 | 0.011 | 4.60E-16 |
| rs7141420 | T | C | 0.617 | 0.024 | 0.003 | 1.23E-14 | 321970 | 0.017 | 0.003 | 5.10E-07 | 212098 | 0.004 | 0.010 | 2.70E-02 |
| rs758747 | C | T | 0.733 | -0.022 | 0.004 | 7.47E-10 | 308688 | -0.007 | 0.004 | 6.30E-02 | 200285 | -0.009 | 0.012 | 5.07E-06 |
| rs7599312 | G | A | 0.708 | 0.022 | 0.003 | 1.17E-10 | 322024 | 0.009 | 0.004 | 1.70E-02 | 212134 | 0.006 | 0.012 | 3.37E-03 |
| rs7899106 | A | G | 0.950 | -0.040 | 0.007 | 2.96E-08 | 321770 | -0.011 | 0.008 | 1.40E-01 | 212004 | -0.009 | 0.024 | 3.19E-02 |
| rs7903146 | T | C | 0.250 | -0.023 | 0.003 | 1.11E-11 | 322130 | -0.003 | 0.004 | 4.30E-01 | 212185 | -0.000 | 0.011 | 9.53E-01 |
| rs929641 | A | G | 0.617 | 0.017 | 0.003 | 1.11E-07 | 322004 | 0.020 | 0.003 | 4.20E-09 | 212102 | -0.015 | 0.011 | 3.58E-16 |
| rs9400239 | C | T | 0.700 | 0.019 | 0.003 | 1.61E-08 | 321988 | 0.015 | 0.004 | 2.10E-05 | 212123 | 0.017 | 0.011 | 7.57E-18 |
| BMI = body mass index, EA = effect allele, EAF = effect allele frequency, LD = linkage disequilibrium, MR = Mendelian randomization, OA = other allele, SE = standard error, WHR = waist-hip-ratio  a) Coefficients are given per SD unit, 4.6 kg/m^2^ and 0.07, for BMI and WHR, respectively, consistent with the Locke (2016) and Shungin (2015) GWAS meta-analyses. For the analyses the coefficients were used on the SD scale for both exposures and rescaled to whole units for BMI and 0.1 units for WHR.  b) Coefficients are given on the logarithmic scale, obtained from a quasi-Poisson regression of hospital admission count on the relevant SNP, using person-years on study as an offset and adjusting for age, sex and the first 40 PCAs | | | | | | | | | | | | | | |

**Figure S1**. Plots for two-sample MR analysis of BMI effect (per SD, with SD_BMI_=4.6) on yearly hospital admission rate in UK Biobank participants of White British ancestry. Effects are shown on the log(rate) scale. A) Cochran’s Q is plotted against Rucker’s Q for each SNP, as calculated in a leave-one-out analysis. Outliers have been visually identified and are labeled. Q-statistics for the model with the full set of 64 SNPs (red) and with outliers removed (61 SNPs, blue) are shown. B) SNP effect on yearly hospital admission count is plotted against the SNP effect on BMI. Fitted lines are displayed for the random effects MR-Egger, penalized weighted median, weighted mode and random effects exact weight IVW models. The outliers identified in plot *A* are shown in blue. * denotes a significant slope.

**BMI**

**A**


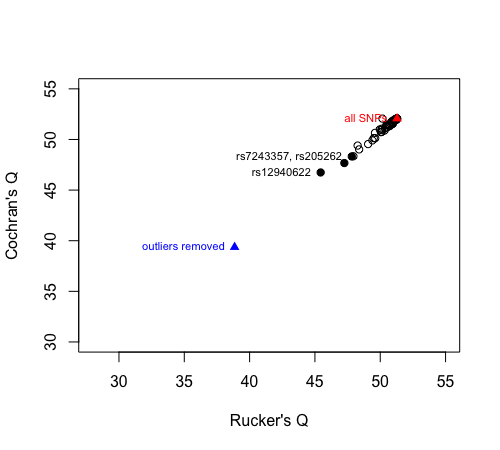


**B**


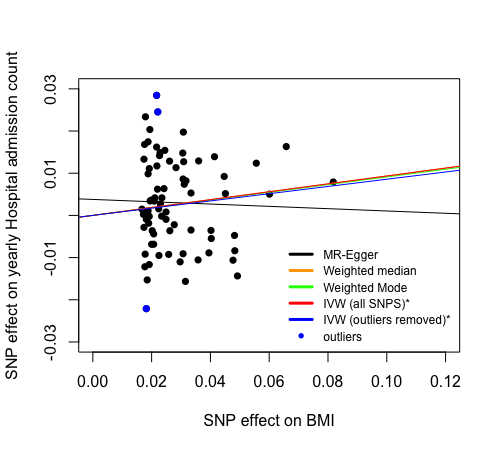


**Figure S2**. Plots for two-sample MR analysis of WHR effect (per SD, with SD_WHR_=0.07) on yearly hospital admission rate in UK Biobank participants of White British ancestry. Effects are shown on the log(rate) scale. A) Cochran’s Q is plotted against Rucker’s Q for each SNP, as calculated in a leave-one-out analysis. Outliers have been visually identified and are labeled. Q-statistics for the model with the full sets of 34 SNPs (red) and with outliers removed (30 SNPs, blue) are shown. B) SNP effect on yearly hospital admission count is plotted against the SNP effect on WHR. Fitted lines are displayed for the random effects MR-Egger, penalized weighted median, weighted mode and random effects exact weight IVW models. The outliers identified in plot *A* are shown in blue. * denotes a significant slope.

**WHR**

**A**


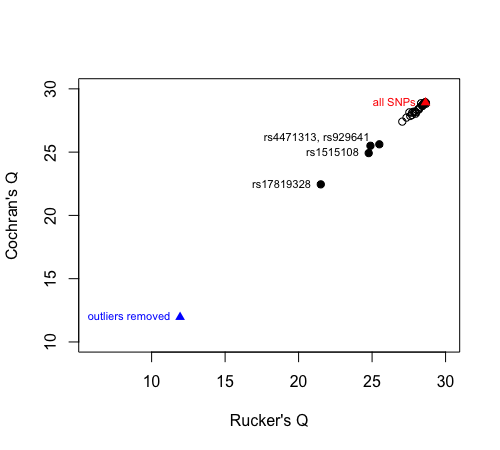


**B**


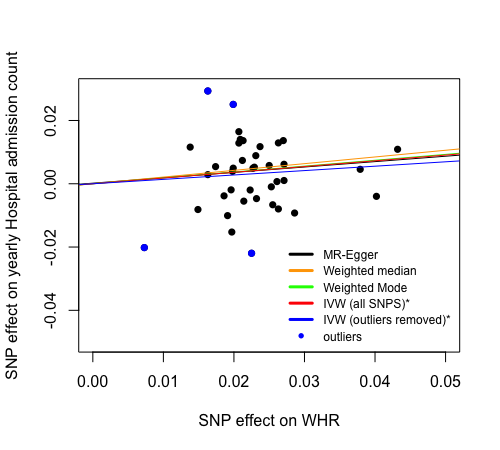


**Figure S3**. Plots for two-sample MR analysis of WHRadjBMI effect (per SD, with SD_WHR_=0.07) on yearly hospital admission rate in UK Biobank participants of White British ancestry. Effects are shown on the log(rate) scale. A) Cochran’s Q is plotted against Rucker’s Q for each SNP, as calculated in a leave-one-out analysis. Outliers have been visually identified and are labeled. Q-statistics for the model with the full set of 45 SNPs (red) and with outliers removed (41 SNPs, blue) are shown. B) SNP effect on yearly hospital admission count is plotted against the SNP effect on WHRadjBMI. Fitted lines are displayed for the random effects MR-Egger, penalized weighted median, weighted mode and random effects exact weight IVW models. The outliers identified in plot *A* are shown in blue. * denotes a significant slope.

**WHRadjBMI**

**A**


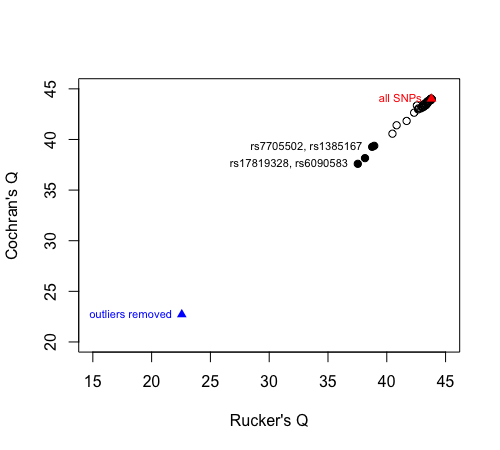


**B**


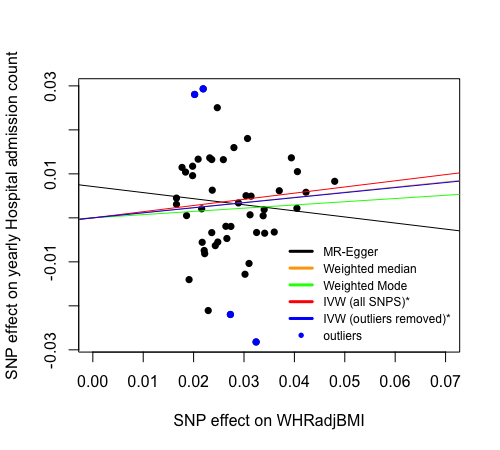

Supplement: Supplementary file 1 — Supplementary material [file mmc1.docx]
